# Meta-transcriptomics indicates biotic cross-tolerance in willow trees cultivated on petroleum hydrocarbon contaminated soil

**DOI:** 10.1186/s12870-015-0636-9

**Published:** 2015-10-12

**Authors:** Emmanuel Gonzalez, Nicholas J. B. Brereton, Julie Marleau, Werther Guidi Nissim, Michel Labrecque, Frederic E. Pitre, Simon Joly

**Affiliations:** Institut de recherche en biologie végétale, University of Montreal, 4101 Sherbrooke E, Montreal, QC H1X 2B2 Canada; Montreal Botanical Gardens, 4101 Sherbrooke E, Montreal, QC H1X 2B2 Canada

**Keywords:** *Salix*, Biomass, Transcriptomics, Meta-transcriptomics, Plant abiotic stress, Plant biotic stress, *Tetranychus*, Crop physiology, Phytoremediation, RNA-seq

## Abstract

**Background:**

High concentrations of petroleum hydrocarbon (PHC) pollution can be hazardous to human health and leave soils incapable of supporting agricultural crops. A cheap solution, which can help restore biodiversity and bring land back to productivity, is cultivation of high biomass yielding willow trees. However, the genetic mechanisms which allow these fast-growing trees to tolerate PHCs are as yet unclear.

**Methods:**

*Salix purpurea* ‘Fish Creek’ trees were pot-grown in soil from a former petroleum refinery, either lacking or enriched with C10-C50 PHCs. De novo assembled transcriptomes were compared between tree organs and impartially annotated without *a priori* constraint to any organism.

**Results:**

Over 45 % of differentially expressed genes originated from foreign organisms, the majority from the two-spotted spidermite,* Tetranychus urticae*. Over 99 % of *T.urticae* transcripts were differentially expressed with greater abundance in non-contaminated trees. Plant transcripts involved in the polypropanoid pathway, including phenylalanine ammonia-lyase (PAL), had greater expression in contaminated trees whereas most resistance genes showed higher expression in non-contaminated trees.

**Conclusions:**

The impartial approach to annotation of the de novo transcriptomes, allowing for the possibility for multiple species identification, was essential for interpretation of the crop’s response treatment. The meta-transcriptomic pattern of expression suggests a cross-tolerance mechanism whereby abiotic stress resistance systems provide improved biotic resistance. These findings highlight a valuable but complex biotic and abiotic stress response to real-world, multidimensional contamination which could, in part, help explain why crops such as willow can produce uniquely high biomass yields on challenging marginal land.

**Electronic supplementary material:**

The online version of this article (doi:10.1186/s12870-015-0636-9) contains supplementary material, which is available to authorized users.

## Background

The ubiquitous use of oil and petroleum products in society has led to localised pollution of land with by-products from petroleum refining, such as aliphatic and polycyclic aromatic hydrocarbons that can be carcinogenic to humans and toxic to most agricultural crops [1]. The number of contaminated sites is thought to be as high as 30,000 across Canada [2], 384,400 across the US [3] and 342,000 across the EU (although *potentially* contaminated sites have been estimated at 2.5 million) [4].

Such large resources of “marginal” land have been recognised as both socially and economically important to rejuvenate and could become more important if predicted increases in the vulnerability of food security, driven by climate change associated weather extremity, are realised [5, 6]. The ability to cultivate efficient and resilient perennial crops on marginal land, thus bypassing the food vs fuel debate and maximising land-use, is essential for the future of agriculture [7]. An emerging approach for cheap and environmentally sustainable land decontamination is phytoremediation, which exploits natural physiological mechanisms of plants to degrade, immobilise and/or selectively uptake contaminants from soil and water [8]. Fast growing short-rotation-coppice willows have emerged as a promising phytoremediation crop, capable of producing high biomass yields (~15 t ha^−1^ year^−1^) on polluted or degraded land [9, 10] as well as an attractive potential lignocellulosic crop [11], which can benefit local biodiversity [12] and does not require high fertiliser application [13]. Some varieties of willow are highly tolerant to organic contaminants such as*:* polycyclic aromatic hydrocarbons (PAHs), polychlorinated biphenyls (PCBs) and petroleum hydrocarbons, and inorganic contaminants such as: As, Cd, Co, Cr, Cu, Hg, Mn, Ni, Pb, Sn and Ti [14, 15]. High biomass yields have also been maintained on land contaminated with high amounts of C10-C50 hydrocarbons, such as that of a petrochemical plant in Varennes, Quebec, (957 mg kg^−1^) [16]. Whilst the physiology of willow has been studied in the presence of common soil contaminants, the molecular mechanisms underpinning this tolerance are still largely unknown.

Several trials have investigated the gene expression or transcriptomic response of willow, and similar crops, to a number of inorganic contaminants applied in isolation, such as: chromium [17], copper [18], zinc [19] and cadmium [20]. Little research, however, has investigated the transcriptomic response of woody crops to organic contamination. Recent research has explored rhizospheric bacteria and their role, hypothesised from expression profiles, in organic contamination tolerance by trees; Yergeau *et al.* [21] found overrepresentation of transcripts implying widespread changes in localised microbial competition. The expression profiles of endophytic bacteria in the above-ground tissues of these trees have been potentially hampered by traditional culturing of bacterial communities, which can limit the potential to identify some of the organisms of interest. However, research has identified some above-ground phytoremediation endophytes; Kang *et al.* [22] isolated a poplar endophyte that can degrade trichloroethylene, an organic contaminant.

To better understand how fast-growing trees can tolerate real-world polluted land, we assessed the changes in expression profiles of willow trees grown using soil from the site of a former petroleum refinery; either contaminated or non-contaminated with high levels of C10-C15 petroleum hydrocarbons. Recent evidence suggests endophytic bacteria are intimately involved in tree stress responses [23–25], and could potentially play an important role in the phytoremediation process. It also seems, as a more general factor to consider, that almost all organisms are involved in complex interdependent relationships as part of a “metaorganism” [26, 27]; we therefore assessed differentially expressed sequences without the limitation of direct mapping or annotation to *Salix purpurea* genome alone, providing the opportunity for an unconstrained view of the origin of sequenced RNA.

## Results

### Tree growth

Composition of contaminated soil from Varennes was highly enriched with C10-C50’s, PCB’s and PAH’s. The specific concentration level of each contaminant was assessed for each pot in the trial. In contaminated pots, C10-C50’s concentration was on average 837.5 mg kg^−1^, PAH’s (collectively) averaged 62.5 mg kg^−1^ and PCB’s averaged 0.2 mg kg^−1^ (Additional file 1: Table S1). The composition of non-contaminated soil, taken from the same site, did not have abundant amounts of these contaminants: C10-C50’s <100 mg kg^−1^, PAH’s <0.1 mg kg^−1^, and PCB’s <0.017 mg kg^−1^. Trees did not show any observable difference in phenotype between treatments over the 6 months of the experiment, this included height which was an average of 223 cm at the time of harvest. Oven dried biomass yields were not significantly different (students *t*-test, *p* = 0.67) at an average of 47.31 g (sd = 8.66) and 45.09 g (sd = 4.48) for non-contaminated and contaminated trees, respectively.

### *De novo* transcriptome assembly and annotation

**Fig. 1:**
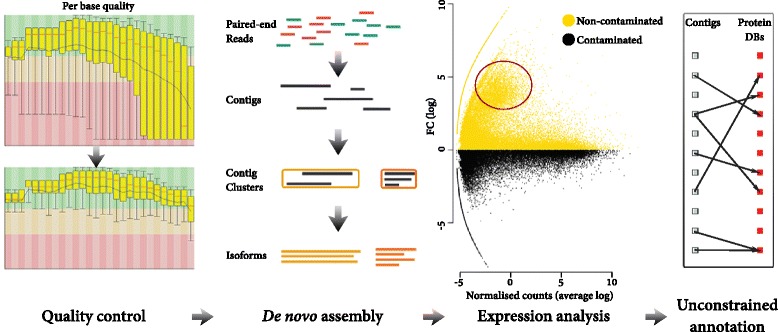
De novo transcriptome assembly and analysis pipeline. *Quality control* - Paired-ends reads are filtered to remove poor quality reads and nucleotides. *De novo assembly* - Transcriptome is assembled *de novo* using Trinity. *Expression analysis* - Mean abundance of all Trinity genes by treatment. Region highlighted in red represents treatment asymmetry in fold change (FC) distribution. *Unconstrained annotation* - Gene annotation pipeline (see Fig. 2)

**Fig. 2:**
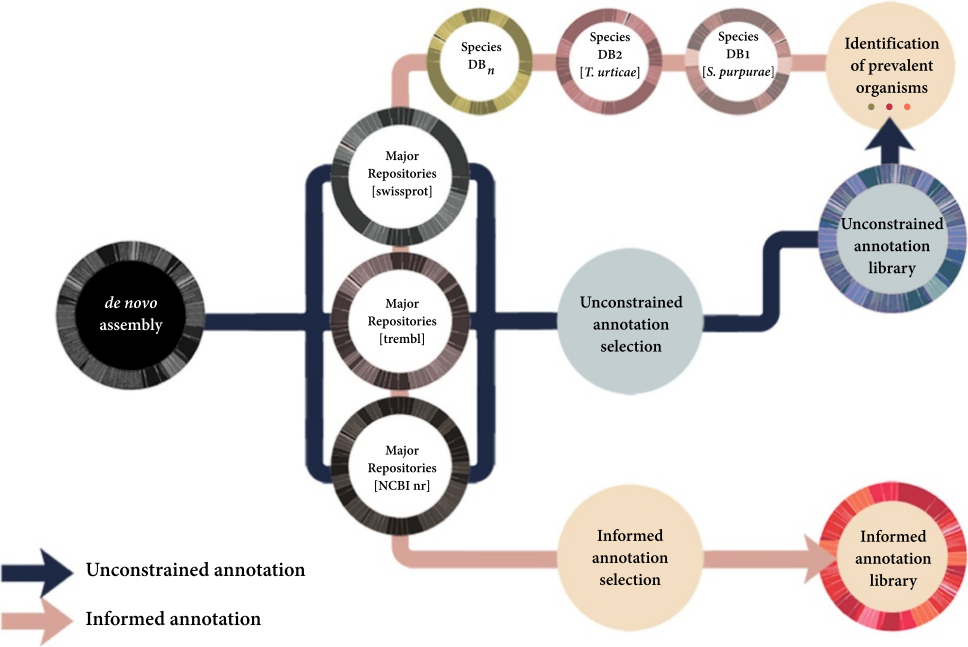
Schematic approach; unconstrained and informed metatranscriptomic annotation. An initial *unconstrained annotation* is aimed at retention of metaorganismal data, allowing the potential for discoveries of system biology. Once informed, selection of annotation from very similar competing blast hits can be given an order of priority, as performed here: <10 % difference in bitscore and protein coverage: i) *S. purperea*, ii) *T. urticae,* iii) SwissProt, iv) Trembl, v) NCBI nr. *DB* database

RNA sequencing was used to estimate transcript abundance for three organs (buds, leaves or stems) sampled from eight trees, four grown on contaminated and four grown on non-contaminated soil, resulting in a total of 24 samples. Depth was a total of 2.4 billion reads, assembled into 424,752 isoforms (1,064,713,007 bp in total) that belonged to 68,191 Trinity genes. N50 of the isoforms was 3,392 bp and the average GC content 39.3 %. At this stage of *de novo* transcriptome analysis the transcripts of non-plant origin are often discarded prior to differential gene expression analysis. Here all assembled transcripts were retained, whatever their origin. A degree of asymmetry was observed within the entire gene expression dataset, with a large group of transcripts substantially upregulated in non-contaminated trees (Fig. 1). Two major species were identified by an initial impartial annotation of DE genes, here termed unconstrained annotation, *S .purpurea* and *T. urticae*. Species-specific sequence databases were added (to Swiss-Prot, TrEMBL and NCBI nr), allowing for a second, final annotation, here termed “informed annotation” (Fig. 2).

### Differentially expressed gene origin

Out of the total 7275 unique genes that were identified as significantly differentially expressed, 6536 were assigned protein identity whilst 739 were classified as unknown (Additional file 2: file S1). Using this annotation approach (Fig. 2), transcripts were best annotated from a total of 138 different organisms.

**Fig. 3:**
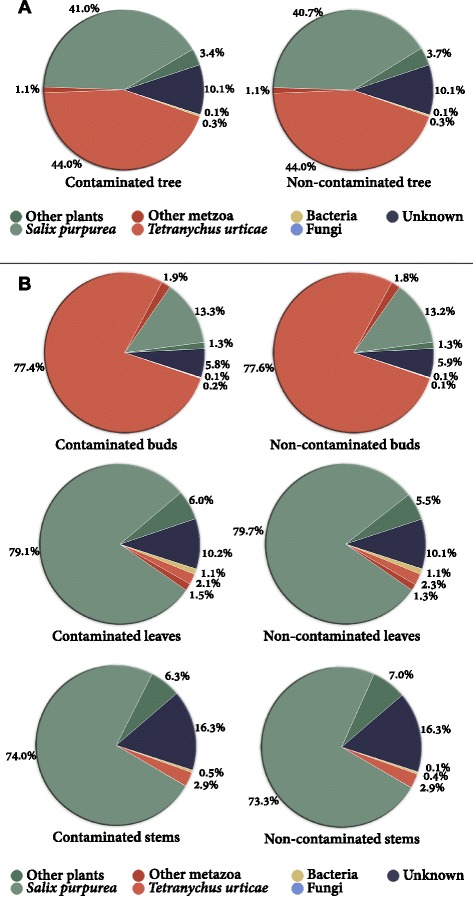
Origin of unique genes differentially expressed due to treatment. **a** The origin of DE transcripts across the entire transcriptome and **b** separated by organ tissue after the best hit was selected from blast querying of *S. purpurea*, *T. urticae*, Swiss-Prot, TrEMBL and NCBI nr protein databases. PPDE >=0.95

The most prominent plant species was *S. purpurea*, as expected, with 41 % of all unique DE genes (Fig. 3a). We also found 0.3 % potential bacterial genes and 0.1 % potential fungal genes. Unexpectedly, a very high number of DE transcripts were best annotated from animal species, with 44 % of all unique DE genes identified as from the polyphagous herbivore *T. urticae,* or two-spotted spidermite.

Large variation in the species origin of DE genes was observed between the different tree organs, however, species composition varied very little between treatments (Fig. 3b). Although most plant DE genes were best annotated from the *S. purpurea* genome (Clone 94006), the likely source of all plant genes being the *S. purpurea* cultivar ‘Fish Creek’ grown here, around 8 % were preferentially annotated (>10 % better identity) from non-*Salix* plant species. The proportion of *S. purpurea* genes within the respective organ transcriptomes remained similar in leaves and stems, with leaves having 79–80 %, and stems having 73-74 %, in contaminated trees and non-contaminated trees respectively (Fig. 3b). Bud DE genes, however, differed markedly from stems and leaves in terms of species of origin. Only 13 % of DE genes in buds were from *S. purpurea*, over 77 % of the transcripts being annotated from *T. urticae*. The number of unique DE genes also varied substantially between organs but remained very similar between treatments; with stems, for example, having roughly four times more diverse *S. purpurea* transcripts than leaves (Additional file 2: file S1). One of the important aspects here was that, even though almost all the unique spidermite contigs were present in buds of both contaminated and non-contaminated trees, the *abundance* was so different (99 % of transcripts were in higher abundance in non-contaminated trees) (fragments per kilobase of transcript per million - fpkm) willow genes would have been highly biased if spidermite RNA was ignored. The importance of identifying as much RNA as possible is clearly of real consequence, not only in terms of the systems biological interactions, but also for the accurate technical quantification willow gene expression.

Gene ontology is often used as a means to reduce complexity of large’omic data to give clues as to trends of physiological response. A very large proportion of differentially expressed plant genes did not have GO (47.2 %), KEGG (96.5 %), KOG (57.3 %) or Panther (41.4 %) classification terms, so caution is taken in relying on partial data. However, a substantial number of genes involved in plant stress responses were differentially expressed, including large overrepresentation of oxidoreduction and defence proteins (Additional file 2: file S1).

### Plant abiotic stress gene expression responses

When plant DE genes were investigated directly, those involved in responses to general and abiotic stress were identified in both contaminated and non-contaminated trees; the most prominent of which were involved in oxidoreduction mechanisms, drought stress and salinity stress responses.

A number of genes involved in reactive oxygen species (ROS) production and ROS scavenging mechanisms were differentially expressed (Additional file 1: Table S2). A cystolic ascorbate peroxidase 1 (APX1) was present in very high abundance in trees grown under both types of treatment (340 fpkm). A number of peroxidases were expressed in very high abundance relative to the contaminated transcriptome as a whole, *SapurV1A.0209s0110.1* and *SapurV1A.1899s0050.1*. A larger number of peroxidases were upregulated, to a lesser degree, in non-contaminated trees. Variation in redox mechanics, identified with genes potentially involved in ROS scavenging, such as glutamate synthase (*SapurV1A.0807s0060.1* and *SapurV1A.0174s0260.1*), were exclusively present in greater abundance in contaminated trees; whereas three OG FeII oxidoreductase gene transcripts, *SapurV1A.0587s0100.1*, *SapurV1A.0006s0890.1* and *SapurV1A.0020s0550.1*, were in greater abundance exclusively in non-contaminated trees. Nine DE gene transcripts with putative roles in drought resistance or response were identified as in greater abundance in contaminated trees. Highly abundant with respect to the transcriptome as a whole was a gene transcript coding for an early-responsive to dehydration protein (*SapurV1A.0030s0160.1*) that presents at an average of 360 fpkm in contaminated trees as well as dehydrin (*SapurV1A.0016s0910.1*), an abiotic responsive group of late embryogenesis abundant proteins, at 120 fpkm.

Genes involved in cell wall construction were differentially expressed between the two treatments; of 78 annotated cell wall polysaccharide genes, 38 had transcripts in higher abundance in contaminated trees and 40 in non-contaminated trees (Additional file 1: Table S3). Notably, cellulose synthase subunit A (*SapurV1A.0027s0400.1*) gene transcripts were in high abundance in contaminated trees. All of the seven DE xyloglucan endotransglycosylase/hydrolase (XET) gene transcripts were in higher abundance in non-contaminated trees. A substantial array of ethylene, auxin, abscisic acid, gibberellin and brassinosteriod, biosynthesis and receptor DE genes were also identified (Additional file 1: Table S4). Ethylene overproducer-like gene transcripts were uniformly in greater abundance in contaminated trees (*SapurV1A.0376s0050.2*, *SapurV1A.3328s0010.1*, *SapurV1A.0376s0050.2* and *SapurV1A.0006s1540.1*) as well as transcripts from a 1-aminocyclopropane-1-carboxylate oxidase (ACO - *SapurV1A.0829s0140.1*) gene that was in very high abundance.

**Fig. 4:**
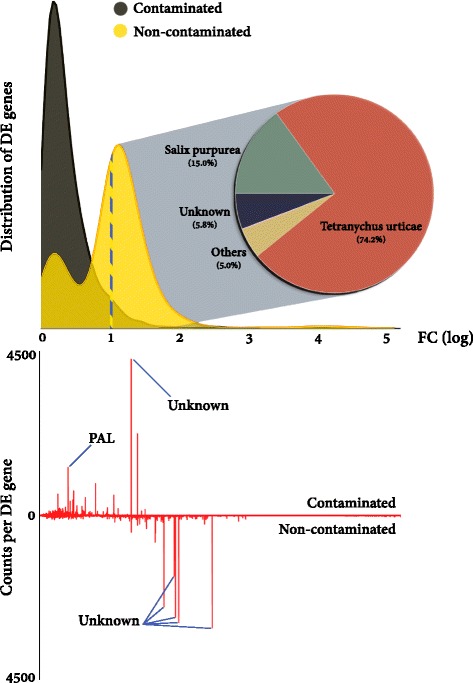
Differentially expressed gene distribution and abundance weighted fold change. Fold change (FC) distribution of DE genes (*top*) per treatment. The origin (organism) of genes with FC increases of >1 (log_10_) in non-contaminated trees is highlighted (dashed line and pie chart). Individual (normalised mean) transcript counts (fpkm) per differentially expressed gene (*bottom*) are segregated by fold change for a weighted view of differential expression. The higher of the two treatments is shown for each DE gene. Major peaks in abundance weighted FC are highlighted and labelled. *PAL* phenylalanine ammonia lyase. PPDE >=0.95

**Fig. 5:**
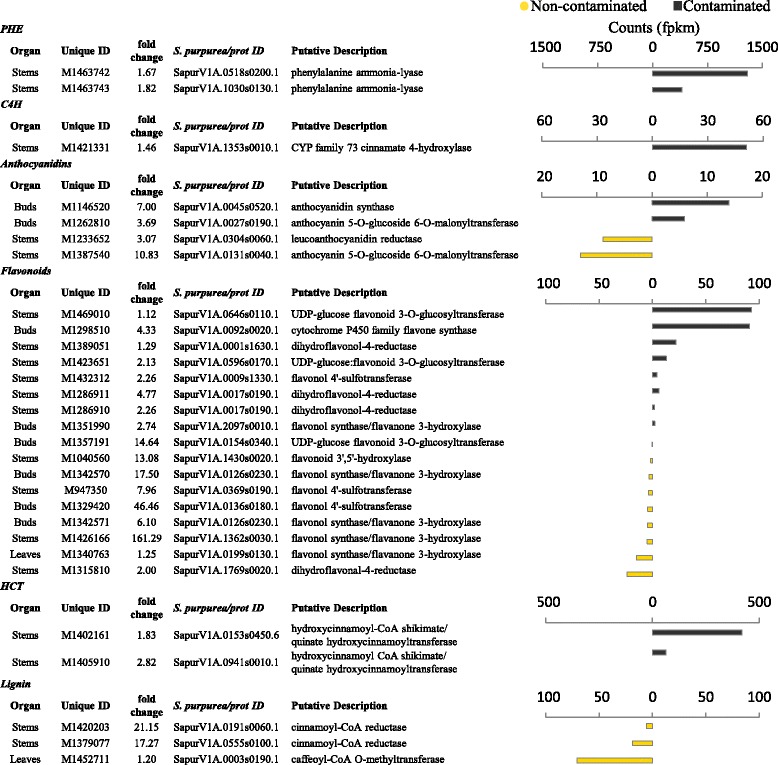
Phenylpropanoid pathway DE genes. Differentially expressed genes functionally classified to the phenylpropanoid pathway. *Salix purpurea*, Swiss-Prot, TrEMBL or NCBI nr. PPDE >=0.95

Both transcript fold change and relative abundance between treatments are potentially, but not necessarily, a valuable means by which to interrogate large transcriptomic datasets for DE genes of interest. A useful way to integrate these two approaches is by using abundance weighted fold change (Fig. 4, Additional file 2: file S1). The transcript with the strongest homology to phenylalanine ammonia lyase (PAL), the third most abundant DE plant transcript in the contaminated trees across all tissues, was the only well annotated transcript to prominently stand out using weighted fold change. Two PAL gene transcripts, with strong homology to PAL in the *S. purpurea* genome, were present in high abundance in the contaminated trees (Fig. 5). The principal route for plant defence metabolite production, the phenylpropanoid pathway, begins with deamination of phenylalanine by PAL. A number of genes downstream of PAL in the polypropanoid pathway were also differentially expressed. Genes involved in production of defence secondary metabolites, such as phenolic glycosides and condensed tannins, were upregulated in both treatments. Notably transcripts from a cytochrome P450 flavone synthesis gene were in high abundance in contaminated trees (*SapurV1A.0092s0020.1*). Genes directly involved in lignin subunit biosynthesis, cinnamoyl-CoA reductase (*SapurV1A.0191s0060.1* and *SapurV1A.0555s0100.1*) and caffeoyl-CoA O-methyltransferase (*SapurV1A.0003s0190.1*) had transcripts consistently in higher abundance in non-contaminated.

### Plant biotic stress gene expression responses

**Fig. 6:**
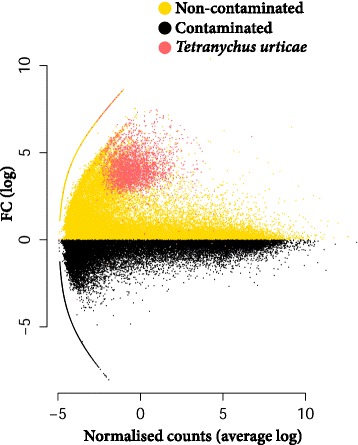
Asymmetry of gene expression in non-contaminated trees. Average fold change (FC) between treatment and transcript count for all assembled genes. Differentially expressed *T. urticae* genes (from all tissues) are highlighted in red. PPDE >=0.95

**Fig. 7:**
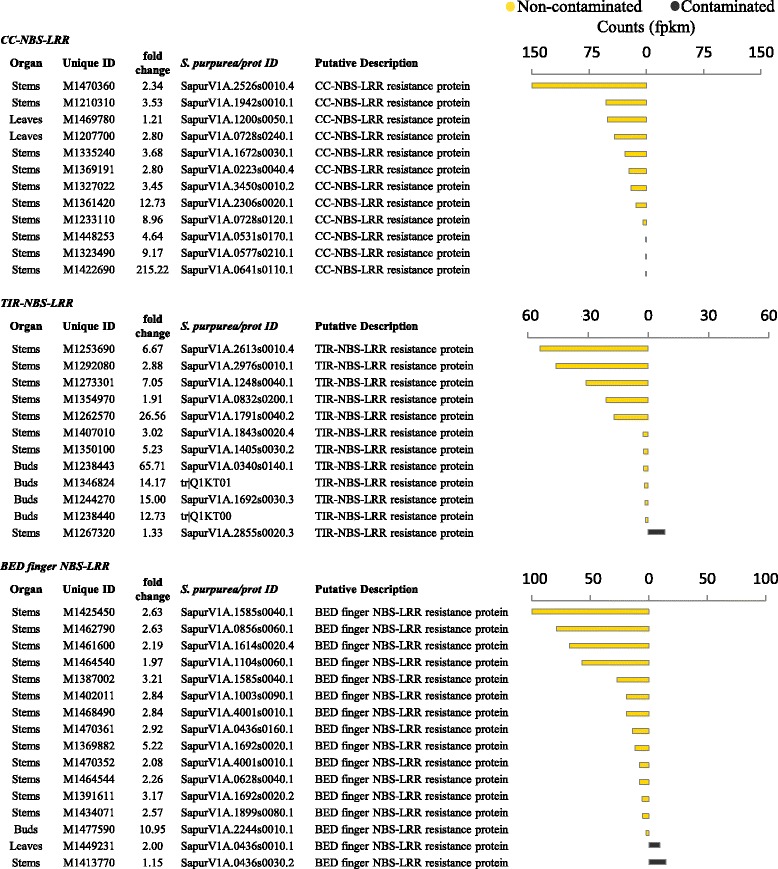
Plant DE resistance genes. Differentially expressed genes functionally classified to biotic resistance. *Salix purpurea*, Swiss-Prot, TrEMBL or NCBI nr, as well as in-house unique identifiers are provided. Direction of differential expression is illustrated graphically with the most abundant counts (fpkm) presented by treatment. PPDE >=0.95

The clearest treatment-specific effect was the very high abundance of non-plant DE gene transcripts in non-contaminated trees. We observed that 44 % of the unique DE transcripts across all tissues were best identified as *T. urticae* genes (when blasted without substantial bias towards any given organism); with the buds having the majority of metazoan sequence, 97 % of which was *T. urticae* (Fig. 3b). *Tetranychus urticae* genes had very stark treatment-specific expression, with 99 % in higher abundance in non-contaminated trees (Figs. 4 and 6). Under the assumption that *T. urticae* preferentially infested non-contaminated trees, we investigated whether *Salix* resistance gene (R-gene) expression reflected an increase in biotic challenge in a treatment-specific manner. Of the R-genes, 12 coiled-coil nucleotide-binding site leucine-rich repeat genes (CC-NBS-LRR) were identified as differentially expressed and were in greater abundance in non-contaminated trees without exception (Fig. 7). Twelve toll-interleukin (TIR-)NB-LRR DE genes were identified with transcripts in greater abundance in non-contaminated trees with one exception (*tr|Q1KT00*_poptr). There were also 16 BED finger NBS-LRR genes identified, 14 with transcripts in greater abundance in non-contaminated trees.

### *Tetranycus urticae* gene expression

**Fig. 8:**
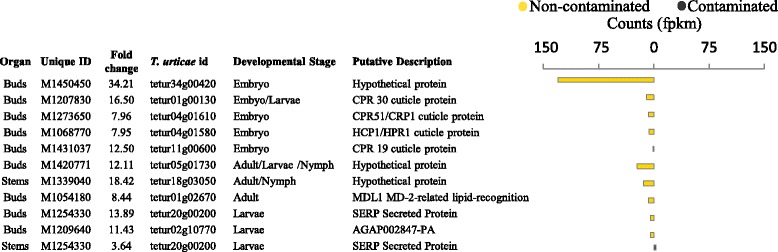
*Tetranychus urticae* developmental stage markers. Differentially expressed genes functionally classified as a developmental stage marker. *T. urticae* and in-house unique identifiers are provided. Direction of differential expression is illustrated with the treatment of highest mean transcript abundance highlighted in bold. PPDE >=0.95

A number of *T. urticae* transcripts whose abundance is thought specific to a given developmental stage were found to be differentially expressed and were in higher abundance in the buds of non-contaminated trees (Fig. 8) but also at high levels compared to the rest of all the DE genes in the non-contaminated tree transcriptome. Three larva specific markers were identified: *tetur20g00200*, *tetur02g10770* and *tetur20g00200* as well as one adult specific marker *tetur01g02670*. Four Embryo specific markers were upregulated: *tetur04g01610*, *tetur04g01580*, *tetur11g00600* and *tetur34g00420*. Only the Embryonic marker *tetur34g00420* was present at the extraordinary high abundance expected as characteristic of a dominant developmental stage.

**Fig. 9:**
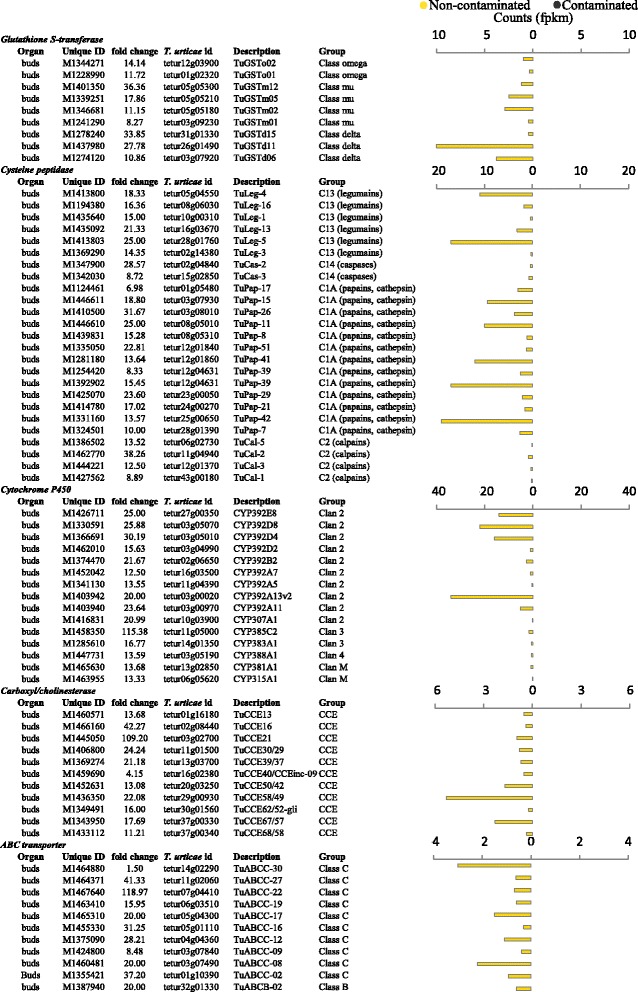
*Tetranychus urticae* DE genes, willow herbivory arsenal. Differentially expressed genes functionally classified to herbivory. *Tetranychus urticae* and in-house unique identifiers are provided. Direction of differential expression is illustrated graphically with the most abundant counts (fpkm) presented by treatment. PPDE >=0.95

Transcripts encoding *T. urticae* detoxification proteins, characteristic of arthropod responses to toxic plant secondary metabolites, were identified as present in high abundance in the non-contaminated trees: these included fifteen cytochrome P450 genes (including clan’s 2, 3 and 4 as well as mitochondrial), nine glutathione S-transferases genes (of classes omega, mu and delta), 11 Carboxyl/cholinesterase genes and 10 ABC C transporters (and 1 ABC B transporter) (Fig. 9). A suite of cysteine peptidases also had transcripts in greater abundance in the buds of non-contaminated trees. These cover the four major classes identified as the spidermite’s proteolytic digesting equipment: papains (C1A), legumains (C13), caspases (C14) and calpains (C2). Transcripts from four C1A papain (*tetur08g05010*, *tetur12g01860*, *tetur12g04631* and *tetur25g00650*) and two C13 legumain genes (*tetur05g04550* and *tetur28g01760*) were present in the highest abundance.

### Microorganism differentially expressed genes

Thirty-two transcripts derived from microorganisms were differentially expressed in response to treatment. Although only 25 % of the transcripts annotated from microorganisms had informative functional description, the taxonomic origin of transcripts can be informative. Six unique transcripts, derived from *Propionibacterium acnes*, were all in greater abundance in buds of non-contaminated trees expressed alongside the very high *T. urticae* gene expression (Additional file 1: Table S5). These included a glycosyl hydrolase (GH65) and a protein with an alpha/beta hydrolase domain. A number of bacteria, such as *Bacillus stratosphericus* and *Klebsiella* sp., were putatively identified as the origin of transcripts in greater abundance in trees cultivated on contaminated soil.

## Discussion

### Willow tolerance of petroleum hydrocarbons

The cultivar ‘Fish Creek’ (*S. purpurea*) has been shown to tolerate high levels of C10-C50 petroleum hydrocarbon soil contamination without substantial losses to biomass yields [16]. The capacity of this crop to tolerate real-world levels of petroleum by-product contamination establishes the potential to utilise and derive value from this type of unused marginal land via biomass and bioproduct production. This tolerance was further demonstrated here by the lack of significant reduction in biomass yield or even any clear, observable phenotypic in pot-grown greenhouse trees established in contaminated soil.

### *De novo* transcriptome assembly and unconstrained annotation

Although effective *de novo* approaches exist for transcriptome assembly, the annotation step is often a bottleneck for non-model organism studies, especially if multiple accessions (or species) are being compared. The complexity of extra-laboratory biological systems, and concepts such as the meta-organism [26, 27], further exacerbates this problem as foreign organisms (such as endophytes) are excluded *ipso facto* from analysis during mapping of RNAseq data to a reference genome or are deliberately discarded as foreign upon annotation. That is, even when a reference genome is available, using it for quantifying gene expression alone by direct mapping can be a risky, or even flawed, approach. Software such as Trinity allows comparison of *de novo* assemblies from different cultivars (or accessions) without bias due to genetic distance from a reference genome. Transcripts can then be annotated by blasting against a reference genome but those assembled transcripts which are not present in the reference genome, such as those unique to an accession, are also preserved and can be annotated independently. Here *de novo* assembly was preferentially utilised even though a single willow cultivar was being assessed and the annotated genome sequence for that species (*S. purpurea* clone 94006) was available. This was due partly to the broad diversity of willow in mind but also because of compelling research performed by Doty *et al.* [22–24, 28] identifying endophytes involved in phytoremediation in *Populus trichocarpa*, indicating treatment-specific variation in expression from foreign organisms is important to maintain and reveal. Any DE transcripts unique to *S. purpurea* cv. ‘Fish Creek’ but not present in *S. purpurea* cv. Clone 94006 (the reference genome) are also retained, an important choice given that 8 % of plant transcripts we identified as better annotated by plants other than this reference *Salix* genome (Fig. 3a). More importantly, over 50 % of the unique transcripts differentially expressed between treatments would have been lost if RNA reads would have been mapped directly to the reference *Salix* genome.

### Plant organ species profiles

The substantial variation in types of non-*salix* species present between the organs suggests that these different tissues represent highly distinctive environments. From a metaorganismal perspective, it would be surprising if the multispecies makeup was constant throughout a plant, as different tissues are hospitable to different organisms. This is supported by the similarity of species (transcript) distribution between treatments within an organ and that very few DE transcripts were shared between organs (412 shared between two organs). Only a very small number of DE genes were unique to treatment within an organ, variation coming from abundance of DE transcripts. Bao *et al.* [29] recently demonstrated that at least 36 % of genes in poplar (which shares close macrosynteny with willow [30]) undergo alternate splicing, analysis of isoform variation was beyond the scope of the work here but such variation would seem likely.

The willow transcriptome exhibited differential expression of genes implying substantial abiotic or biotic stress in both treatments. Plant genes involved in important pathways, such as ROS synthesis and scavenging mechanisms, were present in both treatments. However, differential expression of genes involved in discrete physiological responses could be identified. Using gene ontology analysis of DE genes to reduce complexity, there were broad changes in nucleotide binding, oxidoreduction and defence protein biosynthesis (Additional file 2: file S1); however, whilst used extensively in model species, gene ontology is less powerful in non-model crops as GO, KEGG or Panther terms were not available for many genes (<60 % annotation of unique transcripts). Similar to the problems of mapping reads to the reference *Salix* genome alone, utilising only 60 % of the data runs the risk of being arbitrarily reductionist. Instead, specific gene expression was investigated for these broad categories; in doing so the authors recognise the risks of interpreting phenotype through transcript abundance alone, beyond successful contamination tolerance, and do so with care.

### Plant abiotic stress gene expression

In high biomass yielding crops, such as willow, the vascular cambium represents the majority of cellular division, and therefore, mass of the tree; so strictly regulated stress mechanisms and responses are not unexpected in stem tissue. The involvement of ROS in stress resistance (and many plant physiological processes) is well established [31, 32] as well as, more specifically, in resistance of willow and closely related organisms [33, 34]. In high abundance in both contaminated and non-contaminated trees (significant DE but very low fold change) was APX1 (Additional file 1: Table S2), a central component of the reactive oxygen gene network in the cytosol of *Arabidopsis* leaves (Davletova *et al.* [35]. Intriguingly, APX1 [36, 37], alongside AP2/ERF transcription factors [38], have also been implicated in biotic and abiotic cross-tolerance.

A number of *Salix* genes likely to be involved in detoxification of ROS as well as general response to the stress induced by C10-C50 contamination were differentially expressed and in greater abundance in contaminated tress. These included a number of glutamate synthase genes, glutamate being a constituent of glutathione, an important molecule for oxidative stress tolerance via ROS detoxification [39], as well as a wide number of drought responsive genes implicit in broad abiotic resistance and also associated, in poplar [40], with production of antioxidant phenolic compounds such as kaempferol and catechin.

Major alterations in cell wall construction can be expected between the two treatments as high numbers of cell wall genes were differentially expressed (Additional file 1: Table S3). XET expression showed a clear treatment specific pattern of great abundance in non-contaminated trees. Research performed by Albert *et al.* [41] revealed XET mRNA accumulation in response to biotic stress from the parasite *Cuscuta reflexa*. The same uniform pattern of higher expression in non-contaminated trees was observed in three transcripts encoding two enzymes directly involved in cell wall lignin monomer biosynthesis (Fig. 5), cinnamoyl-CoA reductase and caffeoyl-CoA O-methyltransferase.

A wide spectrum of differentially expressed brassinosteriod, ethylene, abscisic acid and auxin biosynthesis and receptor genes are to be expected in response to extensive (treatment-specific) biotic and abiotic stress. Genes involved in ethylene signalling were strongly differentially expressed with ethylene overproducer-like transcripts present in high abundance in contaminated trees, along with others involved in the ethylene biosynthesis pathway, including S-adenosyl-L-methionine (SAM), 1-aminocyclopropane-1-carboxylate (ACC) and ACC oxidase (ACO). Interestingly, when viewed alongside the very high overexpression of FLA12 in the stems of contaminated trees (Additional file 1: Table S3), a characteristic marker of tension wood tissue in reaction wood producing poplar [42–44] and willow (Brereton, unpublished) trees, an expression profile very similar to tension wood production emerges. Adding to this is a concurrent increase in the abundance of a CesA in stems of contaminated trees as well as the very high abundance of ACO (Additional file 1: Table S4), thought to result in the secondary xylem tissue localisation (or asymmetry) of ethylene biosynthesis from ubiquitous (non-localised) ACC [45]. A link between salinity and drought stress, and the formation of a tension wood has recently been suggested [46, 47] although as an antagonistic response, where tension wood markers such as FLA12 were down-regulated.

One of the most abundant DE transcripts in contaminated trees (the third for plant transcripts) was PAL: representing one of a number of genes involved in the phenylpropanoid-flavonoids pathways known to drive secondary metabolite production in response to abiotic stress [48, 49]. Transcripts encoding proteins at the beginning of the polypropanoid pathway, including PAL and a cinnamate 4-hydroxylase (C4H) (Fig. 5), were strongly upregulated in contaminated trees, perhaps expected due to high contamination and potential drought/osmotic stress (Additional file 1: Table S2) [33, 34, 40, 50–52]. The relationship between abiotic stress induced increases in PAL and C4H with subsequent increases in phenolics, such as catechin, have previously been observed in other crop species such as tea [53], tomato [54], potato [55] and lettuce [56] as well as being well reported in poplar [57]. Although it is common knowledge that large amounts of salicylic acid can be present in willow, a wide spectrum of other phenolic compounds can be present in very high abundance in *Salix* [58–60]. Many of these are induced defence compounds in response to arthropod herbivory in *Salix* [61, 62] and, interestingly, a number of compounds downstream of PAL catalysis have been shown to be highly induced in response to *T. urticae* feeding [63]. This agrees with the potential for cross-tolerance as common phenolic production is elicited by *either* abiotic or biotic stress. Recent evidence implies this is likely as PAL can be directly induced as a defence response to arthropod herbivory [64] in addition to its role in abiotic stress response.

### *Tetranychus urticae* developmental stage expression profile

* Tetranychus urticae* life cycle progresses over four stages: embryo, larval, nymph and then adult [65, 66]*. T. urticae* DE transcripts, 99 % of which were in higher abundance in buds of non-contaminated trees, were screened for the genes identified as markers for specific developmental stages when highly abundant [65]. Clear, very high abundance of *tetur34g00420* gene, one of the 10 embryo markers, suggests an early developmental stage of *T. urticae* at this time (Fig. 8). Larval markers were also present in relatively small abundance and adult markers in medium abundance.

This important treatment-specific interaction between willow and *T. urticae* would have been lost if the de novo assembled transcriptome were annotated using the *S. purpurea* genome alone (or if reads had been mapped to the reference genome). A drawback of such discoveries, based solely on next-generation sequencing data, is that phenotypic data on *T. urticae* infection is challenging to gather post-hoc. It is interesting to note that, if the harvest was indeed performed at an initial phase of embryonic infection, phenotype might have been difficult to recognise without prior knowledge of the interaction. This is because the microscopic eggs need to be deliberately sought and the characteristic webbing, clearly visible in abundance at later infestation stages, may not yet be established.

### Plant biotic stress gene expression

The majority of biotic or disease resistance genes (R-genes) encode nucleotide binding sequence leucine rich repeat proteins in plants [67]. These ancient proteins represent the incredibly diverse and adaptable frontline defence machinery in plants capable of recognising foreign molecules and then eliciting appropriate responses. Recently Yang *et al.* [68] identified 330 NBS-LRR encoding genes in poplar. In similar research Kohler *et al.* [69] identified 119 CC-NBS-LRR, 64 TIR-NBS-LRR and 34 BED-finger-NBS-LRR genes, suggesting a diverse and extensive resistance arsenal is present. As 99 % of *T. urticae* transcripts were in greater abundance in non-contaminated trees, we considered what the plant response to highly increased infestation would be and investigated the differential expression of R-genes between the two treatments. The hypothesis being that a substantial increase of *T. urticae* challenge in non-contaminated trees would induce distinct immune responses.

A very stark pattern of expression was observed; a wide spectrum of intracellular NBS-LRR encoding R-genes were uniformly in greater abundance in non-contaminated, potentially *T. urticae* infected trees (Fig. 7). These included increased abundance of the largest classes of R-gene intracellular receptors; 100 % of the CC-NB-LRR resistance genes were in greater abundance in non-contaminated trees, 92 % of TIR-NB-LRR resistance genes and 88 % of BED finger NB-LRR resistance genes, providing evidence of a more robust biotic resistance response in non-contaminated trees.

### *Tetranychus urticae* herbivory gene expression

Upregulation of classical *T. urticae* detoxification genes was observed from non-contaminated trees (Fig. 9). These genes included glutathione-S-transferases (GST), carboxyl/cholinesterase [70], ABC transporters and a large number of cytochrome P450’s [65]. GST’s can conjugate toxic compounds, such as exuded plant secondary metabolites (ie. anthrocyclins and alkaloids), into glutathione (GSH). Large numbers of ABC transporters have been shown as present in *T. urticae* (at least 103 genes) with the ABC C transporters identified here (which forms a sister clade to multidrug resistance-associated proteins), known to transport neutral conjugates such as GSH [71]. The spidermite’s digestive arsenal used against willow consists predominantly of cysteine proteases [72, 73], this is reflected in the abundant transcripts present in buds of non-contaminated trees of two classes of cysteine proteases: papain (C1A), likely to have a role in the digestive process, and legumain (C13), potentially involved in feeding and digestion of plant material, were numerous and had transcripts in high abundance [73, 74].

There is an established history of increased herbivory resistance in poor nutrient quality and abiotically challenging environments [75, 76], as well as preferential herbivory, from *T. urticae*, of crops with high nutrient content [77, 78]. Without direct assessment of *T. urticae* infestation we cannot conclusively say whether this increase in plant biotic resistance machinery (R-genes) was because of preferential *T. urticae* herbivory of non-contaminated trees or a compromised biotic response in contaminated trees (such as *T. urticae* effector driven suppression of R-genes). We favour the likelihood of willow cross-tolerance, driven by the soil contamination, as *T. urticae* gene expression was so comprehensively upregulated in non-contaminated trees (99 % of genes were in greater abundance). To test this, targeted research in both greenhouse and field environments is required and could have important implications, both for fundamental biology of abiotic and biotic resistance mechanisms as well as for the phytoremediation industry. Additional evidence supporting this hypothesis comes from Grenier *et al.* [16] where, as an independent observation of mature *S. purpurea* ‘Fish Creek’ phenotype grown on the site where contaminated soil came from (Varennes, QC), it was noted that the insect *Tuberolachnus salignus* (large willow aphid) preferentially infested the willows established on non-contaminated over contaminated land.

### Microorganism differentially expressed genes

Whilst little information can be derived regarding microorganism function due to poor annotation description, the type of bacteria present is interesting as the community make-up is thought to drastically change in response to contamination [79]. The most prominent organism in DE transcripts upregulated in the buds of non-contaminated trees, concurrent with 99 % of *T. urticae* transcripts, was *Propionibacterium acnes* (Additional file 1: Table S5). *Propionibacterium acnes* is well known as an endophyte in grapevine [80], *Thlaspi goesingense* [81] and poplar [82]; a less well recognised role is as part of mite bacterial flora. Known to associate with the *Psoroptes ovis* mite [83], *P. acnes* grows in the midgut where it’s thought to be involved in the digestive process. As the bacteria produces propionic acid from carbohydrate, it is of potential interest that one of the DE transcripts was a glycosyl hydrolase (GH65) which could relate to plant cell wall depolymerisation. It would be of interest to establish if any of the microorganisms represented in non-contaminated trees here were directly part of a *T. urticae* salivary bacterial population as such strategies are used, in some cases, to undermine plant defences [84].

Out of the 14 DE transcripts in greater abundance in contaminated trees, a number were associated with microorganisms commonly found in wastewater. *Bacillus stratosphericus* is characterised as a bacteria which inhabits the stratosphere however, many *B. stratosphericus* isolates come from unclean water sources, perhaps reflecting the contaminated soil here. Zhang *et al.* [85] isolated *B. stratosphericus* from river water (Tyne, UK) and groups such as Gupta *et al.* [86], who demonstrated heavy metal remediation properties in *B. stratosphericus,* isolated from soil rich in municipal waste. Klebsiella sp., also in greater abundance in contaminated trees, have been isolated specifically from sludge in wastewater plants [87]. Along these same lines; one of these 14 transcripts characteristic of contaminated trees was best annotated with an unknown sequence isolated from an (arsenic rich) mine drainage bacterial metagenome [88].

### Cross-tolerance potential of *S. purpurea*

If the increased *T. urticae* infestation of non-contaminated trees is accepted from gene expression analysis alone then a number of potential causes related to treatment can be considered. As discussed above, a possible reason may relate to variation in abiotic stress responses as induced by soil contamination. Differential expression of genes involved in specific biotic and abiotic responses were identified between treatments. Although such relationships are often considered as antagonistic there are many examples of *increases* in biotic resistance due to abiotic stress response, frequently achieved through expression of phenolic defence compounds, in *Arabidopsis* [89], tobacco [38], tomato [90], potato [50]. Genes involved in abscisic acid and salicylic acid, hormones involved in complex stress response regulatory networks, were differentially expressed and are presented as potential candidates for such interactions. This “real world” soil presents a disadvantage for unpicking such interactions but grants advantage in retaining some complexity of these crops systems, applicable to the field, and thus, a wealth of gene candidates with hypotheses of potential value to pursue.

A means by which cross-tolerance could be achieved by *S. purpurea* cultivated on contaminated land could be the increase production of compounds downstream in the polypropanoid pathway, such as condensed-tannins, often produced in high abundance in S*alix* [59]. Kao *et al.* [91] demonstrated strong association between PAL expression and condensed tannin accumulation in poplar. If the same mechanisms exist in willow, including the production of extractable compounds such as condensed tannins [92], then further value to the willow phytoremediation system can be established.

The differential expression of genes due to treatment does suggest a complex interplay between abiotic and biotic stress; this needs to be robustly tested before certainty can be established and the authors speculate about such interactions with care. If true, presence of *T. urticae* would compel an extension of the view of tolerant phytoremediating species such as willow; not only do species such as willow find reduced competition from other plant species in these difficult environments but may also benefit, in principle, from the advantage of reduced biotic challenge.

## Conclusions

Over half of the differentially expressed genes would have been discarded if RNA reads were mapped directly to a reference *Salix* genome and, importantly, a crucial factor would have been overlooked with the potential to interact with the entirety of the meta-transcriptomic system. Roughly equal amounts of unique genes from spidermite were identified as from willow. Close to all genes deriving from spidermites had transcripts in greater abundance in non-contaminated trees, which in turn had a suite of plant resistance genes (also) uniformly in greater abundance than in contaminated trees. We propose that abiotic stress responses in contaminated trees, such as increased PAL expression, could interact with spidermite infestation.

It is an ambitious target to mitigate environmental impact of the petrochemical industry by rejuvenating polluted land as well as adding value to that land, or marginal land capitalisation*,* through the production of biomass. In terms of that added value, these results suggest that the very act of tolerating pollution may provide willows with a secondary competitive advantage, facilitating high biomass yields challenging environments, through cross-tolerance against biotic attack.

## Methods

### Experimental design, plant material and soil composition

Soil was collected from the site of a former petrochemical refinery at Varennes, Canada, either enriched in (contaminated) or lacking (non-contaminated) C10-C50 petroleum hydrocarbons (Additional file 1: Table S1). Cuttings of *S. purpurea* cv. ‘Fish Creek’ were sampled as part of a larger split-plot sampling designed consisting of six blocks [21] of which we only investigated the (randomised) contamination effect. The 20 cm cuttings were first established for 8 weeks in conventional potting media before being transfer to the treatment soil in 20 l pots. Six replicates per treatment were sampled for RNAseq (but only four were sequenced downstream). Growth conditions were 16 h 20 °C day and 8 h 18 °C night with excess watering and individual plant pot saucers to reduce leeching. Trees were grown for 6 months before harvesting: leaves, stem and buds. The leaves sampled were from the 5 to 15th fully unfurled leaf from the top of the stem. The term buds here refers to the developing tip of the stems, not axillary buds. Leaves and buds were flash frozen in liquid nitrogen, stem vascular cambium samples were taken, from stem sections towards the base of the stem, by peeling off bark and scraping the inner developing xylem before flash freezing [93, 94].

### RNA extraction, sequencing and quality control

Total RNA was extracted using the modified CTAB protocol [95, 96] with RNA quantity and quality assessed with a BioAnalyser (Agilent). The four best replicates, in terms of potential degradation, were used for downstream RNAseq (selected from the six samples based on highest RNA integrity number - RIN). TrueSeq 100 bp paired-ends libraries were constructed from extracted mRNA (Illumina® TruSeq® RNA Sample Preparation Kit v1, purifying PolyA containing mRNA using poly-Toligo attached magnetic beads before random hexamer pairing for the cDNA synthesis). Samples were sequenced (four per lane) using an Illumina HiSeq 2000 sequencing system. Sequence data was quality controlled using Trimmomatic [97].

### De novo transcriptome assembly and differential expression analysis

Trinity software [98] was used to reconstruct the transcriptome *de novo* using default settings and discarding transcripts <201 bp. Sequences qualified as a “gene” were the union of transcripts similar enough to be considered by Trinity as putative isoforms of the same gene. Bowtie2 [99] software was used to map RNA-seq reads to the *de novo* assembled transcriptome with strict parameters (--no-discordant --no-mixed). A median of 84 % of RNA-Seq reads mapped back to our transcriptome, suggesting the transcriptome correctly represents our data (see Additional file 2: file S1). Raw and normalised transcript abundance was calculated using eXpress [100] with default parameters.

Given the open nature of the annotation we proceeded with these Trinity genes for differential expression analysis instead putative isoforms. It should be noted that these Trinity genes can also summarise allelic variants and duplicate genes and that, ideally, all isoforms should be analysed individually so biological information of greater resolution can be preserved. To assess differential expression, EBSeq [101] was used to identify genes for which transcript abundance had changed significantly between the two experimental conditions for each tissue. EBSeq is less prone to adjust or to discard data due to assumed distribution or FDR (false discovery rate) control [102], exhibiting more consistent behaviour than other DE software when using these real datasets (data not shown). Default parameters were used with 15 iterations for convergence at an FDR of 5 % with significance identified and expressed as posterior probability differential expression (PPDE) greater or equal to 0.95 [101].

We first performed an *unconstrained* annotation of the transcriptome (Fig. 2) using three major protein databases: nr, SwissProt and TrEMBL. This was deliberately performed without assumption as to the origin of sequence. All DE genes sequences passed through each of the three databases using blastx [103] with the following parameters: e-value 1e^−4^, gapopen 11, gapextend 1, word_size 3, matrix BLOSUM62. For each query, up to 20 results were retained to ensure presence of the highest protein similarities without a sole reliance on e-value, unsuitable for comparison of proteins from different databases [104]. Bitscore and protein coverage were used as selection parameters for the best hit from each database and again for selecting the best hit across databases to build the unconstrained annotation library (Fig. 2).

The most prevalent species revealed using unconstrained annotation of DE genes were then identified: in this case being *S. purpurea* and *T. urticae* (the two-spotted spidermite). Recently, the complete genome and annotation of *S. purpurea* genome has been made available on phytozome (*Salix purpurea* v1.0, DOE-JGI, http://phytozome.jgi.doe.gov/pz/portal.html#!info?alias=Org_Spurpurea), drafted by Smart *et al.* (unpublished) in collaboration with the DOE-JGI. The *T. urticae* genome is available at EnsemblMetazoa (http://metazoa.ensembl.org/Tetranychus_urticae/Info/Index) [65]. Once the most prominent organisms had been identified, a second *informed* annotation (Fig. 2) was performed which included these additional species-specific protein databases. Just less than 43.8 % of the final annotation derived from the *T. urticae* database (3193 unique genes), 40.6 % from *S. purpurea* (2960 unique genes), 0.5 % from Swiss-Prot (33 unique genes), 4.5 % from TrEMBL (324 unique genes), 0.4 % from nr (26 unique genes) and 10.2 % unknown (no hit; 739 unique genes).

## Additional files

Additional file 1: Table S1. Soil contaminants. Table S2 Plant stress-related DE genes. Table S3 Plant carbohydrate/cell wall DE genes. Table S4 Plant hormone/signalling DE genes. Table S5 Differentially expressed transcripts from microorganisms. Table S6 Resistance related DE genes (DOCX 111 kb) Additional file 2: File S1. Containing abundance and annotation of 7486 differentially expressed genes. RNAseq data has been deposited in the European Nucleotide Archive (ENA) and can be found via the unique study accession number: PRJEB10287. (XLSX 1320 kb)
