# Supplementary material for: Meta-transcriptomics indicates biotic cross-tolerance in willow trees cultivated on petroleum hydrocarbon contaminated soil
Source: BMC Plant Biol. 2015 Oct 12;15:246. doi: 10.1186/s12870-015-0636-9 (PMC4603587; doi:10.1186/s12870-015-0636-9)
Supplement: Additional file 1: Table S1. — Soil contaminants. Table S2 Plant stress-related DE genes. Table S3 Plant carbohydrate/cell wall DE genes. Table S4 Plant hormone/signalling DE genes. Table S5 Differentially expressed transcripts from microorganisms. Table S6 Resistance related DE genes (DOCX 111 kb) [file 12870_2015_636_MOESM1_ESM.docx]

# Meta-transcriptomics indicates biotic cross-tolerance in willow trees cultivated on petroleum hydrocarbon contaminated soil

Emmanuel Gonzalez^1†^, Nicholas J. B. Brereton^1†^**^*^**, Julie Marleau^1^, Werther G. Nissim^2,^ Michel Labrecque^1,2^, Frederic E. Pitre^1,2^ and Simon Joly^1,2^

1. Institut de recherche en biologie végétale, University of Montreal, 4101 Sherbrooke E, Montreal QC H1X 2B2, Canada.

2. Montreal Botanical Gardens, 4101 Sherbrooke E, Montreal, QC H1X 2B2, Canada.

^†^ These authors contributed equally to the work.

**^*^**Corresponding author: nicholas.brereton@umontreal.ca

**Supplementary Tables**

**Supplementary Table 1 soil contaminants**

Soil was sampled from the former petrochemical refinery site at Varennes, Canada. Twelve samples were taken from the top 30 cm of the soil from contaminated and non-contaminated areas. Samples were pooled for each treatment and analysed by GC-MS.

|  | **Contaminated mg kg^-1^** | **non-contaminated mg kg^-1^** |
| --- | --- | --- |
| Petroleum hydrocarbons C10 to C50 | 837.5 | <100 |
| PCBs (total congeners) | 0.203 | <0.017 |
| **PAHs** |  |  |
| Acenaphthene | 3.2 | <0.1 |
| Acenaphthylene | 1.0 | <0.1 |
| Anthracene | 8.5 | <0.1 |
| Benz[*a*]anthracene | 2.3 | <0.1 |
| Benzo[*a*]pyrene | 0.6 | <0.1 |
| Benzo[*b,j,k*]fluoranthene | 0.7 | <0.1 |
| Benzo[*c*]phenanthrene | 1.2 | <0.1 |
| Benzo[*g,h,i*]perylene | 0.1 | <0.1 |
| Chrysene | 2.7 | <0.1 |
| Dibenz[*a,h*]anthracene | 0.0 | <0.1 |
| Dibenzo[*a,i*]pyrene | 0.0 | <0.1 |
| Dibenzo[*a,h*]pyrene | 0.0 | <0.1 |
| Dibenzo[*a,l*]pyrene | 0.0 | <0.1 |
| 7,12-Dimethylbenz[*a*]anthracene | 0.0 | <0.1 |
| Fluoranthene | 8.0 | <0.1 |
| Fluorene | 1.4 | <0.1 |
| Indeno[1,2,3-*cd*]pyrene | 0.1 | <0.1 |
| 3-Methylcholanthrene | 0.0 | <0.1 |
| Naphthalene | 0.2 | <0.1 |
| Phenanthrene | 13.1 | <0.1 |
| Pyrene | 15.7 | <0.1 |
| 1-Methylnaphthalene | 0.2 | <0.1 |
| 2-Methylnaphthalene | 0.2 | <0.1 |
| 1.3-Dimethylnaphthalene | 1.2 | <0.1 |
| 2,3,5-Trimethylnaphthalene | 2.2 | <0.1 |

**Supplementary Table 2 Plant stress-related DE genes**

Differentially expressed genes functionally classified to redox, drought, abiotic stress or salinity responsive. *Salix purpurea* (SapurV1A), Swiss-Prot (sp|xx), TrEMBL (tr|xx) or NCBI nr, as well as in-house unique identifiers are provided. Direction of differential expression is illustrated with the most abundant treatment highlighted in bold. PPDE >=0.95.

| *Redox* |  |  |  |  |  |  |  |
| --- | --- | --- | --- | --- | --- | --- | --- |
|  |  |  |  |  |  |  | |
| Organ | Unique ID | fold change | Contaminated Mean fpkm | Non-contam Mean fpkm | *S. purpurea/prot ID* | Description | |
| Stems | M1358690 | 1.02 | **343.00** | 337.00 | tr\|B9HQ83 | Cytosolic ascorbate peroxidase | |
| Stems | M1211620 | 1.69 | **270.00** | 160.00 | SapurV1A.0807s0060.1 | glutamate synthase (NADH) | |
| Stems | M1322150 | 3.62 | **170.00** | 47.00 | SapurV1A.0209s0110.1 | Peroxidase | |
| Stems | M1343620 | 1.73 | **140.00** | 81.00 | SapurV1A.1899s0050.1 | Peroxidase | |
| Stems | M1468380 | 1.36 | **120.00** | 88.00 | SapurV1A.0807s0060.1 | glutamate synthase (NADH) | |
| Stems | M1278070 | 1.69 | **120.00** | 71.00 | SapurV1A.1502s0010.1 | hydrogen peroxide induced protein | |
| Buds | M1469260 | 1.43 | **40.00** | 28.00 | SapurV1A.0047s0070.1 | endoplasmic oxidoreductin protein | |
| Stems | M1432271 | 1.94 | **31.00** | 16.00 | SapurV1A.0700s0130.1 | thioldisulphide oxidoreductase DCC | |
| Stems | M1474600 | 2.08 | **27.00** | 13.00 | SapurV1A.0569s0060.1 | oxidoreductase-like protein | |
| Stems | M1463361 | 1.32 | **25.00** | 19.00 | sp\|O05000 | NADH-ubiquinone oxidoreductase chain 2 | |
| Stems | M1281540 | 3.48 | **24.00** | 6.90 | SapurV1A.0261s0280.1 | gibberellin 20-oxidase | |
| Leaves | M1346880 | 1.75 | **21.00** | 12.00 | SapurV1A.0020s0160.1 | gibberellin 2-oxidase | |
| Stems | M1452070 | 1.38 | **18.00** | 13.00 | SapurV1A.1342s0040.2 | GMC oxidoreductase | |
| Buds | M1226950 | 1.63 | **15.00** | 9.20 | SapurV1A.0047s0070.3 | endoplasmic oxidoreductin protein | |
| Stems | M1232941 | 1.67 | **13.00** | 7.80 | SapurV1A.0174s0260.1 | glutamate synthase (NADH) | |
| Leaves | M1259580 | 2.97 | **9.20** | 3.10 | SapurV1A.0428s0120.1 | peroxidase | |
| Stems | M1452291 | 2.27 | **3.40** | 1.50 | sp\|Q04050 | NADH-ubiquinone oxidoreductase chain 4 | |
| Stems | M1259580 | 4.04 | **2.10** | 0.52 | SapurV1A.0428s0120.1 | peroxidase | |
| Stems | M1307960 | 5.14 | **0.72** | 0.14 | SapurV1A.0448s0010.1 | peroxidase | |
| Buds | M1238560 | 5.42 | 0.12 | **0.65** | tr\|B9GJI3 | Oxidoreductase family protein | |
| Stems | M1333940 | 44.44 | 0.02 | **0.80** | SapurV1A.0171s0270.1 | gibberellin 20-oxidase | |
| Stems | M1275830 | 20.73 | 0.04 | **0.85** | SapurV1A.0024s0590.1 | gibberellin 20-oxidase | |
| Buds | M1226680 | 116.67 | 0.01 | **1.40** | SapurV1A.0081s0030.1 | peroxidase | |
| Buds | M1354901 | 21.62 | 0.074 | **1.60** | SapurV1A.1106s0050.1 | gibberellin 2-beta-dioxygenase | |
| Buds | M1012180 | 11.18 | 0.17 | **1.90** | SapurV1A.0016s1290.1 | peroxidase | |
| Stems | M1259800 | 4.76 | 0.42 | **2.00** | SapurV1A.0587s0100.1 | 2OG-Fe(II) oxygenase oxidoreductase | |
| Buds | M1322150 | 20.91 | 0.11 | **2.30** | SapurV1A.0209s0110.1 | peroxidase | |
| Stems | M1293781 | 11.54 | 0.26 | **3.00** | SapurV1A.0396s0150.2 | peroxidase | |
| Buds | M1259580 | 14.09 | 0.22 | **3.10** | SapurV1A.0428s0120.1 | peroxidase | |
| Stems | M1294040 | 5.28 | 0.72 | **3.80** | SapurV1A.0171s0470.1 | NAD(P)H-quinone oxidoreductase | |
| Stems | M1327870 | 8.07 | 0.57 | **4.60** | SapurV1A.0006s0890.1 | 2OG-Fe(II) oxygenase oxidoreductase | |
| Stems | M1242170 | 74.55 | 0.11 | **8.20** | SapurV1A.0222s0050.1 | peroxidase | |
| Leaves | M1282710 | 1.25 | 7.60 | **9.50** | SapurV1A.0352s0060.1 | FAD-dependent oxidoreductase | |
| Leaves | M1393390 | 1.18 | 11.00 | **13.00** | SapurV1A.2901s0020.2 | flavoprotein ubiquinone oxidoreductase | |
| Buds | M1463361 | 1.45 | 11.00 | **16.00** | sp\|O05000 | NADH-ubiquinone oxidoreductase chain 2 | |
| Buds | M1270861 | 25.35 | 0.71 | **18.00** | SapurV1A.0020s0550.1 | 2OG-Fe(II) oxygenase oxidoreductase | |
| Stems | M1410560 | 1.64 | 11.00 | **18.00** | SapurV1A.0093s0540.4 | NADP-dependent oxidoreductase | |
| Stems | M1406743 | 1.63 | 24.00 | **39.00** | tr\|Q5MA12 | NADH-ubiquinone oxidoreductase chain 1 | |
| Leaves | M1448940 | 1.61 | 33.00 | **53.00** | SapurV1A.0750s0180.1 | quinone-oxidoreductase-like protein | |
| Stems | M1468672 | 1.01 | 69.00 | **70.00** | sp\|Q14FF3 | NAD(P)H-quinone oxidoreductase subunit K | |
|  |  |  |  |  |  |  | |
| *Drought* | |  |  |  |  |  | |
| Organ | Unique ID | fold change | Contaminated Mean fpkm | Non-contam Mean fpkm | *S. purpurea/prot ID* | Putative Description | |
| Stems | M1423752 | 1.50 | **360.00** | 240.00 | SapurV1A.0030s0160.1 | early-responsive to dehydration protein | |
| Stems | M1353220 | 1.20 | **120.00** | 100.00 | SapurV1A.0016s0910.1 | dehydrin | |
| Stems | M1408553 | 1.14 | **83.00** | 73.00 | SapurV1A.0857s0230.1 | dehydration-induced protein, putative | |
| Stems | M1423754 | 1.66 | **58.00** | 35.00 | SapurV1A.0030s0160.1 | early-responsive to dehydration protein | |
| Stems | M1380921 | 2.28 | **41.00** | 18.00 | SapurV1A.0586s0150.3 | dehydration-responsive family protein | |
| Stems | M1269450 | 1.00 | **34.00** | 34.00 | SapurV1A.0739s0020.1 | dehydration-induced-like protein | |
| Stems | M1402134 | 3.00 | **4.80** | 1.60 | SapurV1A.2070s0020.1 | drought responsive element-binding protein | |
| Stems | M1209651 | 2.14 | **4.70** | 2.20 | SapurV1A.1241s0160.1 | dehydration-responsive protein RD22 | |
| Buds | M1260590 | 4.75 | **1.90** | 0.40 | SapurV1A.0014s1100.1 | early-responsive to dehydration stress-like | |
| Buds | M1258735 | 23.51 | 0.04 | **0.87** | SapurV1A.0429s0330.1 | early-responsive to dehydration stress-like | |
| Stems | M1355341 | 2.24 | 0.58 | **1.30** | SapurV1A.0580s0090.1 | E3 ubiquitin protein ligase DRIP2, putative | |
| Leaves | M1367883 | 1.17 | 24.00 | **28.00** | SapurV1A.0423s0180.1 | dehydration-responsive element-binding | |
|  |  |  |  |  |  |  | |
| *Abotic TFs* |  |  |  |  |  |  | |
| Organ | Unique ID | fold change | Contaminated Mean fpkm | Non-contam Mean fpkm | *S. purpurea/prot ID* | Putative Description | |
| Stems | M1351940 | 1.28 | **51.00** | 40.00 | SapurV1A.0826s0010.1 | AP2/ERF domain transcription factor | |
| Leaves | M1314910 | 1.73 | **26.00** | 15.00 | SapurV1A.0237s0310.1 | AP2/ERF domain transcription factor | |
| Stems | M1405721 | 2.56 | **8.70** | 3.40 | SapurV1A.0767s0130.1 | AP2/ERF domain transcription factor | |
| Buds | M1196030 | 13.00 | **2.60** | 0.20 | SapurV1A.0282s0070.1 | CBF/DREB1 transcription factor 2 | |
| Stems | M1258380 | 37.04 | 0.03 | **1.00** | SapurV1A.0270s0240.1 | ERF2 transcription factor | |
| Stems | M1099460 | 28.00 | 0.05 | **1.40** | SapurV1A.0006s0870.1 | AP2/ERF domain transcription factor | |
| Buds | M1368621 | 10.63 | 0.160 | **1.70** | SapurV1A.0517s0130.1 | AP2/ERF domain transcription factor | |
| Stems | M1338350 | 73.17 | 0.04 | **3.00** | SapurV1A.0007s0970.1 | AP2/ERF domain transcription factor | |
| Stems | M1290922 | 2.08 | 13.00 | **27.00** | SapurV1A.0079s0190.1 | AP2/ERF domain transcription factor | |
|  |  |  |  |  |  |  | |
| *Salinity* |  |  |  |  |  |  | |
| Organ | Unique ID | fold change | Contaminated Mean fpkm | Non-contam Mean fpkm | *S. purpurea/prot ID* | Putative Description | |
| Stems | M1406761 | 19.09 | 0.22 | **4.20** | SapurV1A.0033s0580.1 | salt stress response/antifungal domain | |
| Leaves | M1391550 | 1.25 | 20.00 | **25.00** | SapurV1A.0957s0090.1 | salt-tolerance protein | |

**Supplementary Table 3 Plant carbohydrate/cell wall DE genes**

Differentially expressed genes functionally classified to cell wall biosynthesis, modificiation or hydrolysis. *S. purpurea* (SapurV1A), Swiss-Prot (sp|xx), TrEMBL (tr|xx) or NCBI nr, as well as in-house unique identifiers are provided. Direction of differential expression is illustrated with the treatment of highest mean transcript abundance highlighted in bold. PPDE >=0.95.

| Organ | Unique ID | fold change | Contaminated Mean fpkm | Non-contam Mean fpkm | *S. purpurea/prot ID* | Putative description |
| --- | --- | --- | --- | --- | --- | --- |
| Buds | M1433090 | 3.08 | **370.00** | 120.00 | SapurV1A.0237s0330.1 | endo-beta-1,4-glucanase |
| Stems | M1463391 | 1.46 | **190.00** | 130.00 | SapurV1A.0027s0400.1 | cellulose synthase A |
| Stems | M1365801 | 1.09 | **74.00** | 68.00 | SapurV1A.0752s0050.1 | 1,3-beta-glucan synthase |
| Stems | M1443370 | 1.14 | **57.00** | 50.00 | SapurV1A.1034s0020.2 | glucan endo-1,3-beta-D-glucosidase |
| Stems | M1431483 | 1.25 | **20.00** | 16.00 | SapurV1A.0419s0300.1 | cellulose synthase-like C1-2 |
| Stems | M1220391 | 2.41 | **2.10** | 0.87 | SapurV1A.1529s0050.1 | cellulose synthase-like |
| Stems | M919130 | 4.47 | **1.70** | 0.38 | SapurV1A.0413s0130.1 | glucan 1,3-beta-glucosidase |
| Buds | M970200 | 14.67 | 0.08 | **1.10** | SapurV1A.0072s0010.1 | beta-glucosidase |
| Stems | M1206790 | 22.22 | 0.54 | **12.00** | SapurV1A.0098s0410.1 | endo-1,4-beta-glucanase |
| Stems | M1373231 | 16.47 | 1.70 | **28.00** | tr\|R7W714 | Cellulose synthase-like E6 |
| Leaves | M1309940 | 1.57 | 83.00 | **130.00** | SapurV1A.0653s0030.1 | alpha-1,4 glucan phosphorylase L isozyme |
| Stems | M1408873 | 1.53 | **46.00** | 30.00 | SapurV1A.0197s0250.1 | xylogalacturonan beta-1,3-xylosyltransferase |
| Stems | M1408070 | 2.07 | **29.00** | 14.00 | SapurV1A.0197s0260.1 | xylogalacturonan beta-1,3-xylosyltransferase |
| Stems | M1411488 | 2.07 | **12.00** | 5.80 | SapurV1A.0809s0070.1 | beta-xylosidase/alpha-L-arabinofuranosidase |
| Buds | M1280130 | 18.06 | 0.03 | **0.56** | SapurV1A.3449s0010.1 | xyloglucan endotransglucosylase/hydrolase |
| Buds | M1260970 | 15.38 | 0.13 | **2.00** | SapurV1A.0049s0070.1 | xyloglucan endotransglucosylase/hydrolase |
| Stems | M1325520 | 28.57 | 0.08 | **2.40** | SapurV1A.0542s0160.1 | xyloglucan endotransglycosylase/hydrolase |
| Buds | M1215810 | 8.16 | 0.38 | **3.10** | SapurV1A.5351s0010.1 | xyloglucan endotransglycosylase hydrolase |
| Stems | M1260970 | 26.52 | 0.23 | **6.10** | SapurV1A.0049s0070.1 | xyloglucan endotransglucosylase/hydrolase |
| Stems | M1344400 | 14.42 | 0.52 | **7.50** | SapurV1A.0907s0010.1 | xyloglucan specific endoglucanase inhibitor |
| Stems | M1418671 | 7.31 | 1.30 | **9.50** | SapurV1A.0821s0120.1 | xyloglucan endotransglycosylase hydrolase |
| Stems | M1325522 | 30.93 | 0.97 | **30.00** | SapurV1A.0198s0180.1 | xyloglucan endotransglycosylase/hydrolase |
| Stems | M1459374 | 1.84 | **160.00** | 87.00 | SapurV1A.0616s0030.2 | beta-galactosidase |
| Stems | M1389517 | 1.53 | **75.00** | 49.00 | SapurV1A.0240s0050.1 | beta-galactosidase |
| Stems | M1317072 | 2.17 | **50.00** | 23.00 | SapurV1A.0904s0080.1 | beta-galactosidase |
| Leaves | M1399700 | 2.63 | **42.00** | 16.00 | SapurV1A.0192s0320.2 | galactinol synthase |
| Stems | M1295881 | 1.77 | **39.00** | 22.00 | SapurV1A.0114s0480.1 | polygalacturonase |
| Stems | M1210890 | 1.06 | **17.00** | 16.00 | SapurV1A.0537s0060.1 | galactoside 2-alpha-L-fucosyltransferase |
| Buds | M1245340 | 4.38 | **14.00** | 3.20 | SapurV1A.0014s0240.1 | polygalacturonase |
| Stems | M1243420 | 2.05 | **8.00** | 3.90 | SapurV1A.0104s0250.1 | polygalacturonase QRT3 |
| Stems | M1468222 | 2.00 | **6.80** | 3.40 | SapurV1A.1150s0120.1 | galactosyltransferase |
| Stems | M1298650 | 3.59 | **6.10** | 1.70 | SapurV1A.0365s0170.2 | beta-galactosidase |
| Stems | M1207951 | 2.24 | **5.60** | 2.50 | SapurV1A.0369s0390.1 | galactoside 2-alpha-L-fucosyltransferase |
| Buds | M1451703 | 1.59 | **5.10** | 3.20 | SapurV1A.0548s0160.2 | beta-galactosidase |
| Stems | M1207952 | 1.96 | **4.50** | 2.30 | SapurV1A.0369s0390.1 | galactoside 2-alpha-L-fucosyltransferase |
| Buds | M1243420 | 2.00 | **1.50** | 0.75 | SapurV1A.0104s0250.1 | polygalacturonase QRT3 |
| Buds | M1436681 | 2.39 | 0.18 | **0.43** | SapurV1A.1368s0080.1 | rhamnogalacturonate lyase |
| Stems | M1359670 | 2.33 | 0.21 | **0.49** | SapurV1A.0973s0040.2 | beta-galactosidase |
| Buds | M1395300 | 7.82 | 0.11 | **0.86** | SapurV1A.0169s0160.1 | polygalacturonase |
| Buds | M1418080 | 7.96 | 0.49 | **3.90** | SapurV1A.0269s0180.1 | galactinol synthase |
| Buds | M1348750 | 5.93 | 0.81 | **4.80** | SapurV1A.0004s0700.1 | rhamnogalacturonate lyase-like |
| Stems | M1201810 | 13.33 | 0.36 | **4.80** | SapurV1A.0094s0270.1 | galactose oxidase |
| Buds | M1338010 | 1.69 | 2.90 | **4.90** | SapurV1A.1343s0030.1 | beta-galactosidase |
| Stems | M1363922 | 1.38 | 7.10 | **9.80** | SapurV1A.0057s0260.1 | rhamnogalacturonate lyase |
| Stems | M1363981 | 11.25 | 1.60 | **18.00** | SapurV1A.0069s0050.1 | galactinol synthase |
| Stems | M1418083 | 11.11 | 1.80 | **20.00** | SapurV1A.0269s0180.1 | galactinol synthase |
| Stems | M1391281 | 1.57 | 14.00 | **22.00** | SapurV1A.0409s0140.22 | rhamnogalacturonate lyase-like |
| Buds | M1418083 | 6.76 | 3.40 | **23.00** | SapurV1A.0269s0180.1 | galactinol synthase |
| Stems | M1399701 | 32.05 | 0.78 | **25.00** | SapurV1A.0269s0180.1 | galactinol synthase |
| Stems | M1460290 | 3.33 | 9.00 | **30.00** | SapurV1A.1145s0010.1 | beta-galactosidase |
| Buds | M1266321 | 13.33 | 3.00 | **40.00** | SapurV1A.3501s0010.1 | galactose oxidase |
| Buds | M1371050 | 1.41 | 32.00 | **45.00** | SapurV1A.0161s0100.1 | galactosyltransferase |
| Stems | M1374153 | 2.60 | 50.00 | **130.00** | SapurV1A.0432s0180.1 | beta-galactosidase |
| Stems | M1434935 | 2.24 | **13.00** | 5.80 | SapurV1A.0487s0190.1 | GDP-mannose 4,6-dehydratase |
| Stems | M1449223 | 1.45 | **12.00** | 8.30 | SapurV1A.0744s0140.2 | mannan endo-1,4-beta-mannosidase |
| Stems | M1425581 | 1.00 | **12.00** | 12.00 | SapurV1A.0351s0190.1 | GDP-fucose O-fucosyltransferase |
| Stems | M1431041 | 1.22 | **10.00** | 8.20 | SapurV1A.0268s0070.1 | GDP-fucose O-fucosyltransferase |
| Stems | M1414504 | 1.88 | **8.10** | 4.30 | SapurV1A.1176s0050.1 | GDP-mannose pyrophosphorylase |
| Stems | M1356762 | 2.33 | **2.80** | 1.20 | SapurV1A.0058s0440.2 | GDP-fucose O-fucosyltransferase |
| Stems | M1442110 | 1.78 | **71.00** | 40.00 | SapurV1A.0521s0110.1 | GH 28 |
| Buds | M1441452 | 1.31 | **42.00** | 32.00 | SapurV1A.0256s0240.1 | secondary cell wall-related GT8 |
| Stems | M1464271 | 1.44 | **39.00** | 27.00 | SapurV1A.0654s0010.1 | UDP-glucosyltransferase |
| Stems | M1312540 | 2.40 | **36.00** | 15.00 | SapurV1A.0176s0030.1 | glycosyltransferase |
| Buds | M1459994 | 1.67 | **20.00** | 12.00 | SapurV1A.1160s0070.3 | GH17 |
| Buds | M1130361 | 1.79 | **12.00** | 6.70 | SapurV1A.0262s0310.1 | UDP-glycosyltransferase |
| Stems | M1343830 | 2.94 | **10.00** | 3.40 | SapurV1A.0228s0110.1 | glycosyltransferase |
| Stems | M1391070 | 4.50 | **7.20** | 1.60 | SapurV1A.0067s0630.1 | GH1 |
| Stems | M1371122 | 1.27 | **6.20** | 4.90 | SapurV1A.2030s0010.1 | UDP-glycosyltransferase |
| Buds | M1291820 | 2.88 | **4.90** | 1.70 | SapurV1A.0643s0020.1 | UDP-glucosyltransferase |
| Buds | M1254750 | 5.77 | **0.15** | 0.03 | SapurV1A.0901s0010.1 | UDP-glucosyltransferase |
| Buds | M1176901 | 19.44 | 0.07 | **1.40** | SapurV1A.0645s0110.1 | UDP-glucose glucosyltransferase |
| Stems | M1476220 | 1.98 | 0.91 | **1.80** | SapurV1A.0237s0210.1 | UDP-glucosyltransferase |
| Buds | M1279150 | 9.60 | 0.25 | **2.40** | SapurV1A.0124s0560.1 | UDP-glucosyltransferase |
| Stems | M1379912 | 9.53 | 0.43 | **4.10** | SapurV1A.0626s0020.4 | GH18 |
| Stems | M1429192 | 23.10 | 0.29 | **6.70** | SapurV1A.0359s0290.1 | glycosyl hydrolase |
| Stems | M1363820 | 2.24 | 4.90 | **11.00** | SapurV1A.0001s0770.1 | glycosyltransferase |
| Stems | M1320370 | 5.20 | 2.50 | **13.00** | tr\|B9H1S9 | GH1 |
| Stems | M1291080 | 2.50 | 6.40 | **16.00** | SapurV1A.0720s0040.1 | UDP-glucosyltransferase |
| Stems | M1431080 | 1.54 | 13.00 | **20.00** | SapurV1A.0153s0400.1 | UDP-glucosyltransferase |
| Stems | M1464432 | 8.80 | 2.50 | **22.00** | SapurV1A.0139s0010.1 | UDP-glucosyltransferase |
| Stems | M1455420 | 12.38 | 2.10 | **26.00** | SapurV1A.1414s0030.1 | UDP-glucose:glucosyltransferase |
| Stems | M1345891 | 42.19 | 0.64 | **27.00** | SapurV1A.2851s0010.1 | UDP-glucosyltransferase |
| Stems | M1386400 | 21.11 | 1.80 | **38.00** | SapurV1A.1324s0060.1 | UDP-glucosyltransferase |
|  |  |  |  |  |  |  |
| *FLAs* |  |  |  |  |  |  |
| Stems | M1440571 | 2.67 | **640.00** | 240.00 | SapurV1A.0216s0260.1.p | FLA12 |
| Stems | M1189130 | 2.35 | **120.00** | 51.00 | SapurV1A.0209s0060.1.p | FLA13 |
| Stems | M1399570 | 2.62 | **17.00** | 6.50 | SapurV1A.0036s0660.1.p | FLA17 |
| Buds | M1128880 | n/a | 0.000 | **0.67** | SapurV1A.0216s0270.1.p | FLA15 |
| Buds | M1366030 | 80.00 | 0.020 | **1.60** | SapurV1A.0604s0140.1.p | FLA15 |
| Buds | M1245171 | 39.08 | 0.087 | **3.40** | SapurV1A.0240s0070.1.p | FLA15 |
| Buds | M1432062 | 13.10 | 0.840 | **11.00** | SapurV1A.0433s0050.1.p | FLA15 |

**Supplementary Table 4 Plant hormone/signalling DE genes**

Differentially expressed genes functionally classified to hormone biosynthesis or perception. *S. purpurea* (SapurV1A), Swiss-Prot (sp|xx), TrEMBL (tr|xx) or NCBI nr, as well as in-house unique identifiers are provided. Direction of differential expression is illustrated with the treatment of highest mean transcript abundance highlighted in bold. PPDE >=0.95.

| *Calcium signalling* | |  |  |  |  |  |
| --- | --- | --- | --- | --- | --- | --- |
| Organ | Unique ID | fold change | Contaminated Mean fpkm | Non-contam Mean fpkm | *S. purpurea/prot ID* | Putative description |
| Stems | M1334022 | 1.94 | **350.00** | 180.00 | SapurV1A.0083s0490.1 | calcium ion-binding protein |
| Stems | M1419172 | 1.28 | **230.00** | 180.00 | SapurV1A.0198s0050.1 | calcium-transporting ATPase |
| Stems | M1448261 | 1.10 | **22.00** | 20.00 | SapurV1A.0277s0290.1 | calcium-dependent kinase |
| Stems | M1442744 | 0.78 | **14.00** | 18.00 | SapurV1A.0823s0130.1 | autoinhibited calcium ATPase |
| Stems | M1331986 | 2.21 | **6.40** | 2.90 | SapurV1A.1150s0010.1 | calcium-binding EF-hand protein |
| Stems | M1290040 | 2.30 | **5.30** | 2.30 | SapurV1A.0084s0110.1 | calcium-binding protein CML16 |
| Stems | M1387750 | 71.43 | 0.02 | **1.50** | SapurV1A.0014s0760.1 | calcium-binding protein |
| Stems | M1274770 | 6.07 | 0.28 | **1.70** | SapurV1A.0994s0030.1 | calcium ion-binding protein |
| Stems | M1177540 | 12.00 | 0.20 | **2.40** | SapurV1A.0840s0060.1 | calcium-binding EF-hand protein |
| Stems | M1104070 | 35.14 | 0.07 | **2.60** | SapurV1A.1276s0010.1 | C2 calcium/lipid-binding and phosphoribosyltransferase |
| Stems | M1424290 | 2.08 | 1.30 | **2.70** | SapurV1A.0698s0070.1 | calcium-transporting ATPase 2 |
| Stems | M1412840 | 15.88 | 0.34 | **5.40** | SapurV1A.0006s1470.1 | calcium-transporting ATPase |
| Stems | M1300180 | 1.93 | 2.90 | **5.60** | SapurV1A.0297s0140.1 | calcium homeostasis regulator CHoR1 |
| Buds | M1301460 | 8.26 | 0.690 | **5.70** | SapurV1A.0729s0220.1 | calmodulin |
| Stems | M1464291 | 2.61 | 3.10 | **8.10** | SapurV1A.0001s0710.1 | C2 calcium/lipid-binding phosphoribosyltransferase-like |
| Stems | M1129020 | 1.86 | 4.90 | **9.10** | SapurV1A.0907s0040.3 | calcium-dependent lipid-binding (CaLB domain) |
| Buds | M1065070 | 9.44 | 1.800 | **17.00** | SapurV1A.0010s0670.1 | calmodulin-like protein |
| Leaves | M1423240 | 1.05 | 21.00 | **22.00** | SapurV1A.0045s0670.1 | calcium-dependent kinase |
| Stems | M1469472 | 1.42 | 19.00 | **27.00** | SapurV1A.0515s0090.3 | calcium-dependent kinase |
| Stems | M1411411 | 2.07 | 15.00 | **31.00** | SapurV1A.1005s0050.1 | calcium-dependent kinase |
| Stems | M1065070 | 91.49 | 0.47 | **43.00** | SapurV1A.0010s0670.1 | calmodulin-like protein |
| Stems | M1371121 | 1.77 | 30.00 | **53.00** | SapurV1A.0030s0360.1 | calcium-dependent lipid-binding domain protein |
| Leaves | M1426612 | 1.31 | 45.00 | **59.00** | SapurV1A.0208s0140.1 | calmodulin-binding family protein |
|  |  |  |  |  |  |  |
|  | |  |  |  |  |  |
| *abacisic acid* | |  |  |  |  |  |
| Organ | Unique ID | fold change | Contaminated Mean fpkm | Non-contam Mean fpkm | *S. purpurea/prot ID* | Putative description |
| Buds | M1373170 | 3.42 | **27.000** | 7.90 | SapurV1A.0041s0270.1 | CYP family 707 ABA 8'-hydroxylase |
| Stems | M1415507 | 2.11 | **20.00** | 9.50 | SapurV1A.0151s0060.1 | abscisic acid receptor |
| Stems | M1393322 | 1.24 | **57.00** | 46.00 | SapurV1A.0167s0060.1 | ABA responsive element-binding factor |
| Buds | M1366482 | 1.56 | 6.300 | **9.80** | SapurV1A.0377s0090.1 | abscisic acid receptor PYL8 |
| Leaves | M1311850 | 1.94 | 6.20 | **12.00** | SapurV1A.0001s0310.1 | abscisic acid receptor PYL4 |
|  |  |  |  |  |  |  |
|  | |  |  |  |  |  |
| *Brassinosteroid* | |  |  |  |  |  |
| Organ | Unique ID | fold change | Contaminated Mean fpkm | Non-contam Mean fpkm | *S. purpurea/prot ID* | Putative description |
| Stems | M1180840 | 2.08 | **0.27** | 0.13 | SapurV1A.0162s0370.1 | brassinosteroid insensitive 1-associated receptor kinase |
|  |  |  |  |  |  |  |
|  | |  |  |  |  |  |
| *Gibberellin* | |  |  |  |  |  |
| Organ | Unique ID | fold change | Contaminated Mean fpkm | Non-contam Mean fpkm | *S. purpurea/prot ID* | Putative description |
| Stems | M1281540 | 3.48 | **24.00** | 6.90 | SapurV1A.0261s0280.1 | gibberellin 20-oxidase |
| Leaves | M1346880 | 1.75 | **21.00** | 12.00 | SapurV1A.0020s0160.1 | gibberellin 2-oxidase |
| Stems | M1333940 | 44.44 | 0.02 | **0.80** | SapurV1A.0171s0270.1 | gibberellin 20-oxidase |
| Stems | M1275830 | 20.73 | 0.04 | **0.85** | SapurV1A.0024s0590.1 | gibberellin 20-oxidase |
| Buds | M1354901 | 21.62 | 0.074 | **1.60** | SapurV1A.1106s0050.1 | gibberellin 2-beta-dioxygenase |
|  |  |  |  |  |  |  |
|  |  |  |  |  |  |  |
| *Auxin* |  |  |  |  |  |  |
| Organ | Unique ID | fold change | Contaminated Mean fpkm | Non-contam Mean fpkm | *S. purpurea/prot ID* | Putative description |
| Stems | M1383340 | 2.00 | **280.00** | 140.00 | SapurV1A.0014s0740.1 | auxin induced-like protein |
| Stems | M1279920 | 2.00 | **260.00** | 130.00 | SapurV1A.0326s0090.1 | auxin responsive protein |
| Stems | M1343600 | 1.76 | **65.00** | 37.00 | SapurV1A.0014s0750.1 | auxin induced-like protein |
| Stems | M1385011 | 1.89 | **53.00** | 28.00 | SapurV1A.0657s0050.1 | auxin response factor |
| Stems | M1404203 | 1.48 | **31.00** | 21.00 | SapurV1A.0198s0240.1 | auxin-responsive family protein |
| Stems | M1385010 | 1.45 | **16.00** | 11.00 | SapurV1A.0518s0120.1 | auxin response factor |
| Stems | M1402503 | 2.67 | **12.00** | 4.50 | SapurV1A.1086s0130.1 | auxin-responsive family protein |
| Stems | M1359242 | 1.87 | **8.80** | 4.70 | SapurV1A.0389s0310.1 | SAUR-like auxin-responsive protein |
| Stems | M1359241 | 2.53 | **4.80** | 1.90 | SapurV1A.0389s0310.1 | SAUR-like auxin-responsive protein |
| Buds | M1349601 | 2.60 | **3.900** | 1.50 | SapurV1A.0267s0160.1 | auxin efflux carrier PIN1 protein |
| Stems | M1290221 | 9.00 | **3.60** | 0.40 | SapurV1A.0696s0070.2 | dormancy/auxin associated protein |
| Stems | M1441100 | 1.70 | **3.40** | 2.00 | SapurV1A.0478s0100.1 | auxin efflux carrier family protein |
| Buds | M1122281 | 4.10 | **2.500** | 0.61 | SapurV1A.0025s0560.1 | auxin responsive protein |
| Stems | M1233770 | 2.64 | **0.74** | 0.28 | SapurV1A.0622s0020.1 | auxin-binding protein ABP19a |
| Buds | M1326870 | 4.78 | **0.220** | 0.05 | SapurV1A.2967s0020.1 | auxin-induced protein 5NG4 |
| Stems | M1252660 | 7.50 | 0.07 | **0.54** | SapurV1A.1640s0100.1 | auxin-responsive family protein |
| Buds | M1244190 | 10.00 | 0.130 | **1.30** | SapurV1A.0625s0090.1 | auxin-induced protein 5NG4 |
| Buds | M1398610 | 5.88 | 0.340 | **2.00** | SapurV1A.0003s0260.1 | auxin-induced protein 5NG4 |
| Stems | M1348771 | 14.80 | 0.25 | **3.70** | SapurV1A.0728s0080.4 | auxin efflux carrier family protein |
| Stems | M1349120 | 9.50 | 0.40 | **3.80** | SapurV1A.0553s0060.1 | auxin-independent growth promoter protein |
| Stems | M1350270 | 11.88 | 0.32 | **3.80** | SapurV1A.0210s0060.1 | auxin-induced protein 5NG4 |
| Buds | M1290221 | 14.48 | 0.290 | **4.20** | SapurV1A.0696s0070.2 | dormancy/auxin associated protein |
| Stems | M1271832 | n/a | 0.00 | **6.70** | SapurV1A.0321s0300.2 | auxin-induced protein 5NG4 |
| Stems | M1271831 | 44.12 | 0.17 | **7.50** | SapurV1A.0321s0300.2 | auxin-induced protein 5NG4 |
| Stems | M1378172 | 2.50 | 4.40 | **11.00** | SapurV1A.1265s0070.1 | auxin influx transporter |
| Stems | M1410210 | 1.60 | 7.50 | **12.00** | SapurV1A.1257s0090.3 | auxin response factor |
| Stems | M1457490 | 2.83 | 4.60 | **13.00** | SapurV1A.0378s0240.1 | auxin-induced protein 5NG4 |
| Stems | M1374880 | 18.00 | 1.00 | **18.00** | SapurV1A.0666s0030.1 | auxin-induced protein 5NG4 |
| Stems | M1400130 | 26.67 | 0.90 | **24.00** | SapurV1A.1141s0050.1 | auxin efflux carrier component |
| Stems | M1452541 | 1.69 | 29.00 | **49.00** | SapurV1A.0030s0180.1 | auxin response factor 6 |
| Stems | M1402634 | 2.06 | 32.00 | **66.00** | SapurV1A.0147s0210.2 | auxin response factor |
| Stems | M1467250 | 1.56 | 90.00 | **140.00** | SapurV1A.0357s0020.3 | auxin efflux carrier PIN1 protein |
|  |  |  |  |  |  |  |
|  | |  |  |  |  |  |
| *Ethylene* | |  |  |  |  |  |
| Organ | Unique ID | fold change | Contaminated Mean fpkm | Non-contam Mean fpkm | *S. purpurea/prot ID* | Putative description |
| Stems | M1337690 | 3.94 | **630.00** | 160.00 | SapurV1A.0829s0140.1 | 1-aminocyclopropane-1-carboxylate oxidase |
| Stems | M1413930 | 1.09 | **120.00** | 110.00 | SapurV1A.0376s0050.2 | ethylene overproducer-like protein |
| Stems | M1271691 | 2.34 | **110.00** | 47.00 | SapurV1A.3328s0010.1 | ethylene overproducer-like protein |
| Stems | M1354710 | 1.59 | **89.00** | 56.00 | SapurV1A.0093s0190.1 | S-adenosylmethionine-dependent methyltransferase |
| Stems | M1080511 | 1.79 | **77.00** | 43.00 | SapurV1A.0376s0050.2 | ethylene overproducer-like protein |
| Stems | M1470130 | 1.75 | **77.00** | 44.00 | SapurV1A.0006s1540.1 | ethylene overproducer-like protein |
| Stems | M1427171 | 1.57 | **66.00** | 42.00 | SapurV1A.0465s0190.2 | S-adenosylmethionine-dependent methyltransferase |
| Buds | M1362630 | 2.31 | **37.000** | 16.00 | SapurV1A.0332s0160.1 | S-adenosyl-L-methionine decarboxylase |
| Stems | M1179050 | 2.31 | **3.00** | 1.30 | SapurV1A.0223s0250.1 | S-adenosyl-L-methionine-dependent methyltransferase |
| Buds | M937430 | 7.18 | **0.560** | 0.08 | SapurV1A.4215s0010.1 | 1-aminocyclopropane-1-carboxylate oxidase |
| Stems | M1262671 | 10.99 | 0.07 | **0.78** | SapurV1A.0087s0280.1 | 1-aminocyclopropane-1-carboxylate synthase |
| Buds | M1179050 | 4.76 | 0.170 | **0.81** | SapurV1A.0223s0250.1 | S-adenosyl-L-methionine-dependent methyltransferase |
| Buds | M1129770 | 10.77 | 0.130 | **1.40** | SapurV1A.0132s0310.1 | ethylene-responsive transcription factor |
| Buds | M1122790 | 11.33 | 0.150 | **1.70** | SapurV1A.0054s0450.1 | S-adenosyl-L-methionine:salicylic acid carboxyl methyltransferase |
| Stems | M1055090 | 12.00 | 0.15 | **1.80** | SapurV1A.0021s0980.1 | ethylene-responsive transcription factor |
| Stems | M1087680 | 23.64 | 0.11 | **2.60** | SapurV1A.0816s0060.1 | ethylene-responsive transcription factor |
| Buds | M1367900 | 7.78 | 0.360 | **2.80** | SapurV1A.0007s0900.1 | 1-aminocyclopropane-1-carboxylate oxidase |
| Stems | M1087681 | 18.82 | 0.17 | **3.20** | SapurV1A.0816s0060.1 | ethylene-responsive transcription factor |
| Buds | M1420110 | 6.49 | 0.570 | **3.70** | SapurV1A.0233s0370.1 | ethylene-responsive transcription factor |
| Buds | M1277670 | 1.78 | 4.600 | **8.20** | SapurV1A.0292s0180.2 | ethylene receptor |
| Stems | M1452476 | 1.70 | 8.80 | **15.00** | SapurV1A.0208s0240.2 | ethylene-responsive kinase Le-CTR1 |
| Stems | M1338730 | 1.78 | 9.00 | **16.00** | SapurV1A.0049s0080.2 | S-adenosylmethionine-dependent methyltransferase |
| Buds | M1467611 | 1.38 | 13.000 | **18.00** | SapurV1A.0007s0810.1 | ethylene receptor |
| Stems | M1129770 | 30.56 | 0.72 | **22.00** | SapurV1A.0132s0310.1 | ethylene-responsive transcription factor |
| Buds | M1444362 | 1.12 | 42.000 | **47.00** | SapurV1A.0110s0420.1 | ethylene receptor |

**Supplementary Table 5 Differentially expressed transcripts from microorganisms**

Differentially expressed genes from microorganisms. Swiss-Prot (sp|xx), TrEMBL (tr|xx) or NCBI nr, as well as in-house unique identifiers are provided. Direction of differential expression is illustrated with the most abundant treatment highlighted in bold. PPDE >=0.95.

| Organ | Unique ID | fold change | Contaminated Mean fpkm | Non-contam Mean fpkm | subjectid | Subject description | Orgainism |
| --- | --- | --- | --- | --- | --- | --- | --- |
| Stems | Momp1469770 | 15.71 | **4400.00** | 280.00 | tr\|K2RWR9_MACPH | Chromo domain-like protein | Macrophomina phaseolina |
| Stems | Momp1467130 | 10.56 | **190.00** | 18.00 | tr\|D6GPH6_9ENTR | Putative uncharacterized protein | Klebsiella sp. 1_1_55 |
| Stems | Momp1442350 | 7.50 | **120.00** | 16.00 | tr\|U4N0L9LOTM | Uncharacterized protein | Ruminiclostridium thermocellum |
| Stems | Momp1355461 | 9.46 | **35.00** | 3.70 | tr\|C1HUN6_9ESCH | Uncharacterized protein | Escherichia sp. 3_2_53FAA |
| Stems | Momp1411224 | 7.93 | **23.00** | 2.90 | tr\|C1HUN1_9ESCH | Uncharacterized protein | Escherichia sp. 3_2_53FAA |
| Stems | Momp1227760 | 12.05 | **8.80** | 0.73 | gi\|490001652 | hypothetical protein | Mycobacterium tuberculosis |
| Stems | Momp1418760 | 8.91 | **8.20** | 0.92 | gi\|545012559 | hypothetical protein | Peptoclostridium difficile |
| Stems | Momp1409381 | 12.74 | **7.90** | 0.62 | tr\|M5RIP4_9BACI | Lipoprotein | Bacillus stratosphericus |
| Stems | Momp1369052 | 6.42 | **7.70** | 1.20 | tr\|D2YVG0_VIBMI | Uncharacterized protein | Vibrio mimicus VM573 |
| Stems | Momp1406050 | 5.69 | **7.40** | 1.30 | gi\|496024205 | hypothetical protein | Streptomyces sp. SPB74 |
| Stems | Momp1390430 | 88.31 | **6.80** | 0.08 | tr\|E2S3A1_9CORY | Uncharacterized protein | Corynebacterium pseudogenitalium |
| Stems | Momp1354230 | 3.05 | **6.70** | 2.20 | tr\|A5Z511_9FIRM | Uncharacterized protein | Eubacterium ventriosum |
| Stems | Momp1374380 | 8.19 | **5.90** | 0.72 | tr\|B1SBJ3_9STRE | Uncharacterized protein | Streptococcus infantarius |
| Stems | Momp1338691 | 6.14 | **5.10** | 0.83 | tr\|E6PDN8_9ZZZZ | Uncharacterized protein | mine drainage metagenome |
| Leaves | Momp1470230 | 9.52 | 0.00 | **0.02** | tr\|D4HDI8_PROAS | ATP-dependent helicase HrpA | Propionibacterium acnes |
| Leaves | Momp1470160 | 10.53 | 0.00 | **0.02** | tr\|E4H989_PROAA | AMP-binding enzyme | Propionibacterium acnes |
| Leaves | Momp1470250 | 7.02 | 0.01 | **0.04** | tr\|F9Z0J5_PROAA | DNA polymerase I | Propionibacterium acnes |
| Leaves | Momp1470080 | 9.53 | 0.00 | **0.04** | tr\|D1YDZ8_PROAA | ATP-dependent chaperone protein | Propionibacterium acnes |
| Leaves | Momp1470140 | 11.89 | 0.00 | **0.04** | tr\|D3MAP9_PROAA | Glycosyl hydrolase family 65 | Propionibacterium acnes |
| Leaves | Momp1469930 | 7.12 | 0.01 | **0.05** | tr\|E4F615_PROAA | Hydrolase, alpha/beta domain protein | Propionibacterium acnes |
| Buds | Momp1461991 | 17.50 | 0.05 | **0.91** | tr\|U9TCL0_RHIID | Uncharacterized protein | Rhizophagus irregularis |
| Buds | Momp1225211 | 21.82 | 0.06 | **1.20** | tr\|E1NMK5_9LACO | Amino acid permease | Lactobacillus iners |
| Buds | Momp1371841 | 7.22 | 0.18 | **1.30** | tr\|E3DAP6_GARV3 | DNA-binding helix-turn-helix protein | Gardnerella vaginalis |
| Buds | Momp1330879 | 11.67 | 0.12 | **1.40** | tr\|I4M645_GARVA | Glutamate--tRNA ligase | Gardnerella vaginalis |
| Leaves | Momp1348733 | 12.94 | 0.17 | **2.20** | gi\|515998198 | hypothetical protein | Vibrio vulnificus |
| Buds | Momp1393980 | 16.00 | 0.15 | **2.40** | tr\|A1D8H5_NEOFI | Putative uncharacterized protein | Neosartorya fischeri |
| Buds | Momp1367040 | 22.94 | 0.17 | **3.90** | tr\|M3A7A3_MYCFI | Uncharacterized protein | Mycosphaerella fijiensis |
| Buds | Momp1231740 | 57.69 | 0.13 | **7.50** | tr\|F9XGJ5_MYCGM | Uncharacterized protein | Mycosphaerella graminicola |
| Buds | Momp1324071 | 115.38 | 0.08 | **9.00** | tr\|E0XQM4_9GAMM | Putative uncharacterized protein | gamma proteobacterium |
| Buds | Momp1354230 | 19.80 | 0.49 | **9.70** | tr\|A5Z511_9FIRM | Uncharacterized protein | Eubacterium ventriosum |
| Buds | Momp1461061 | 12.20 | 0.82 | **10.00** | tr\|D1RK09_LEGLO | Putative uncharacterized protein | Legionella longbeachae |
| Stems | Momp1442390 | n/a | 0.00 | **14.00** | tr\|J3QHX4_PUCT1 | Uncharacterized protein | Puccinia triticina |
|  |  |  |  |  |  |  |  |

**Supplementary Table 6 Resistance related DE genes**

Differentially expressed genes potentially involved in biotic resistance. *S. purpurea* (SapurV1A), Swiss-Prot (sp|xx), TrEMBL (tr|xx) or NCBI nr, as well as in-house unique identifiers are provided. Direction of differential expression is illustrated with the treatment of highest mean transcript abundance highlighted in bold. PPDE >=0.95.

| *pathogenesis-related* | |  |  |  |  |  |
| --- | --- | --- | --- | --- | --- | --- |
| Organ | Unique ID | fold change | Contaminated Mean fpkm | Non-contam Mean fpkm | *S. purpurea/prot ID* | Putative description |
| Stems | M1368670 | 67.65 | 0.34 | **23.00** | SapurV1A.0012s0860.1 | pathogenesis-related protein Bet V I |
| Buds | M1368670 | 59.38 | 0.32 | **19.00** | SapurV1A.0012s0860.1 | pathogenesis-related protein Bet V I |
| Stems | M1306500 | 6.38 | 1.30 | **8.30** | SapurV1A.0279s0060.1 | pathogenesis-related protein 1a |
| Stems | M1306530 | 13.60 | 0.25 | **3.40** | SapurV1A.5963s0010.1 | pathogenesis-related protein |
| Stems | M918230 | 43.75 | 0.05 | **2.10** | SapurV1A.0735s0040.1 | pathogenesis-related protein Bet V I |
| Stems | M921390 | 2.18 | **37.00** | 17.00 | SapurV1A.1296s0030.1 | pathogenesis-related protein Bet V I |
|  | |  |  |  |  |  |
| *Disease resistance* | |  |  |  |  |  |
| Organ | Unique ID | fold change | Contaminated Mean fpkm | Non-contam Mean fpkm | *S. purpurea/prot ID* | Putative description |
| Stems | M1302560 | 139.24 | 0.01 | **1.10** | SapurV1A.0017s0110.1 | disease resistance protein RPM1 |
| Stems | M1329960 | n/a | 0.00 | **4.90** | tr\|M8D5G3 | Disease resistance protein RPM1 |
| Stems | M1468811 | 50.71 | 0.14 | **7.10** | tr\|Q84X58 | NBS-type putative resistance protein |
| Stems | M1342420 | 62.50 | 0.12 | **7.50** | tr\|M7ZNP2 | Disease resistance protein RGA2 |
| Stems | M1432530 | n/a | 0.00 | **14.00** | tr\|M8D5G3 | Disease resistance protein RPM1 |
| Stems | M1342120 | 3.18 | **1.40** | 0.44 | SapurV1A.0051s0380.1 | NBS-LRR type disease resistance protein |
| Leaves | M1468811 | 13.14 | **0.92** | 0.07 | tr\|Q84X58 | NBS-type putative resistance protein |
|  |  |  |  |  |  |  |
| *MDR, ABC transporters* | | |  |  |  |  |
| Organ | Unique ID | fold change | Contaminated Mean fpkm | Non-contam Mean fpkm | *S. purpurea/prot ID* | Putative description |
| Stems | M1362263 | 1.30 | **61.00** | 47.00 | SapurV1A.0046s0110.1 | multidrug/pheromone exporter, ABC |
| Stems | M1402613 | 1.19 | **32.00** | 27.00 | SapurV1A.1064s0110.1 | multidrug/pheromone exporter, ABC |
| Stems | M1402302 | 3.45 | **1.90** | 0.55 | SapurV1A.0438s0120.1 | multidrug/pheromone exporter, ABC |
| Buds | M1414673 | 8.37 | 0.086 | **0.72** | SapurV1A.0009s0920.1 | MDR, ABC transporter |
| Stems | M1316071 | 2.58 | 0.62 | **1.60** | SapurV1A.0035s0850.2 | MDR, ABC transporter |
| Stems | M1422545 | 38.10 | 0.06 | **2.40** | SapurV1A.2000s0010.2 | MDR, ABC transporter |
| Stems | M1422544 | 50.91 | 0.11 | **5.60** | SapurV1A.2000s0010.1 | MDR, ABC transporter |
| Stems | M1401410 | 263.33 | 0.03 | **7.90** | SapurV1A.1189s0020.1 | MDR, ABC transporter |
| Stems | M1452300 | 4.89 | 1.80 | **8.80** | SapurV1A.0155s0280.1 | MDR, ABC transporter |
| Stems | M1250680 | 116.67 | 0.12 | **14.00** | SapurV1A.0454s0050.1 | multidrug/pheromone exporter, ABC |
| Stems | M1250681 | 25.00 | 0.92 | **23.00** | SapurV1A.0454s0050.1 | multidrug/pheromone exporter, ABC |
|  | |  |  |  |  |  |
| *Serine/Threonine, LRR kinase* | | |  |  |  |  |
| Organ | Unique ID | fold change | Contaminated Mean fpkm | Non-contam Mean fpkm | *S. purpurea/prot ID* | Putative description |
| Stems | M1326350 | 2.11 | **120.00** | 57.00 | SapurV1A.0005s0910.1 | LRR disease resistance protein |
| Stems | M1423600 | 1.39 | **79.00** | 57.00 | SapurV1A.0160s0060.1 | Serine/Threonine-kinase |
| Buds | M1466021 | 1.61 | **58.00** | 36.00 | SapurV1A.0661s0010.1 | Serine/Threonine-kinase |
| Stems | M1460150 | 1.70 | **56.00** | 33.00 | SapurV1A.1752s0050.1 | Serine/Threonine-kinase |
| Stems | M1406130 | 1.08 | **41.00** | 38.00 | SapurV1A.0022s0480.1 | Serine/Threonine kinase PBS1 |
| Stems | M1227450 | 2.53 | **38.00** | 15.00 | SapurV1A.0118s0090.1 | Serine/Threonine-kinase |
| Stems | M1355660 | 1.29 | **31.00** | 24.00 | SapurV1A.0240s0160.1 | serine/threonine-protein phosphatase |
| Stems | M1318072 | 1.30 | **30.00** | 23.00 | SapurV1A.0995s0040.2 | Serine/Threonine-kinase |
| Buds | M1426532 | 1.62 | **21.00** | 13.00 | SapurV1A.0236s0260.1 | LRR receptor-like kinase |
| Stems | M1430250 | 1.40 | **21.00** | 15.00 | SapurV1A.0802s0080.1 | LRR receptor-like kinase plant |
| Buds | M1469140 | 1.76 | **9.00** | 5.10 | SapurV1A.1281s0010.1 | serine/threonine protein phosphatase 6 |
| Stems | M1439800 | 1.11 | **7.80** | 7.00 | SapurV1A.1070s0150.1 | LRR receptor-like kinase |
| Buds | M1389141 | 1.52 | **7.30** | 4.80 | SapurV1A.0536s0090.1 | LRR receptor-like Serine/Threonine-kinase |
| Stems | M1354110 | 1.27 | **5.60** | 4.40 | SapurV1A.0363s0020.1 | LRR receptor-like kinase |
| Stems | M1395562 | 3.80 | **3.80** | 1.00 | SapurV1A.1347s0010.1 | Serine/Threonine-kinase |
| Stems | M1469610 | 1.94 | **3.50** | 1.80 | SapurV1A.1051s0030.1 | Serine/Threonine-kinase |
| Stems | M1420731 | 1.83 | **3.30** | 1.80 | SapurV1A.1474s0040.1 | LRR receptor-like kinase |
| Buds | M1438522 | 4.35 | **3.00** | 0.69 | SapurV1A.0207s0230.1 | Serine/Threonine-kinase WNK-like |
| Buds | M992820 | 4.04 | **2.30** | 0.57 | SapurV1A.1648s0060.1 | LRR-like Serine/Threonine-kinase ERL1 |
| Leaves | M1308840 | 2.13 | **1.70** | 0.80 | SapurV1A.0429s0180.1 | Serine/Threonine-kinase |
| Stems | M1406203 | 7.50 | **1.50** | 0.20 | SapurV1A.0167s0030.1 | Serine/Threonine-kinase |
| Buds | M1393360 | 2.74 | **0.96** | 0.35 | SapurV1A.3383s0010.1 | Serine/Threonine-kinase |
| Buds | M1129521 | 3.74 | **0.71** | 0.19 | SapurV1A.0153s0420.1 | LRR receptor-like Serine/Threonine-kinase |
| Buds | M1433124 | 4.88 | 0.04 | **0.20** | SapurV1A.0192s0110.3 | LRR receptor-like kinase |
| Stems | M1253801 | 7.78 | 0.04 | **0.28** | SapurV1A.0348s0220.1 | LRR receptor-like kinase |
| Stems | M1422791 | 6.62 | 0.07 | **0.45** | SapurV1A.0364s0300.1 | serine/threonine protein phosphatase 6 |
| Buds | M998370 | 8.09 | 0.11 | **0.89** | SapurV1A.0680s0080.1 | Serine/Threonine-kinase |
| Buds | M1467550 | 10.00 | 0.12 | **1.20** | SapurV1A.1038s0050.1 | Serine/Threonine-kinase |
| Stems | M1245540 | 9.38 | 0.16 | **1.50** | SapurV1A.0055s0200.1 | Serine/Threonine-kinase |
| Buds | M1283160 | 9.41 | 0.17 | **1.60** | SapurV1A.0005s1020.1 | Serine/Threonine-kinase |
| Stems | M1246800 | 9.55 | 0.22 | **2.10** | SapurV1A.0106s0150.1 | Serine/Threonine-kinase |
| Buds | M1254521 | 23.00 | 0.10 | **2.30** | SapurV1A.0935s0010.1 | Serine/Threonine-kinase |
| Stems | M1212530 | 10.00 | 0.29 | **2.90** | SapurV1A.0112s0200.1 | Serine/Threonine-kinase |
| Stems | M1412520 | 12.31 | 0.26 | **3.20** | SapurV1A.1914s0030.1 | LRR receptor-like kinase |
| Buds | M1402551 | 3.08 | 1.20 | **3.70** | SapurV1A.0995s0060.1 | Serine/Threonine-kinase |
| Stems | M1128890 | 4.10 | 1.00 | **4.10** | SapurV1A.0019s0660.2 | Serine/Threonine-kinase TOR |
| Stems | M1346661 | 2.17 | 2.40 | **5.20** | SapurV1A.0762s0180.1 | LRR receptor-like kinase |
| Buds | M1359590 | 1.64 | 3.90 | **6.40** | SapurV1A.0053s0390.1 | LRR receptor-like kinase |
| Buds | M1432573 | 20.29 | 0.35 | **7.10** | SapurV1A.0157s0010.2 | LRR receptor-like kinase |
| Leaves | M1439800 | 1.22 | 5.90 | **7.20** | SapurV1A.1070s0150.1 | LRR receptor-like kinase |
| Stems | M1232040 | 228.13 | 0.03 | **7.30** | SapurV1A.0020s0580.2 | LRR receptor-like Serine/Threonine-kinase |
| Buds | M1188760 | 5.43 | 1.40 | **7.60** | SapurV1A.0097s0210.1 | Serine/Threonine kinase |
| Stems | M1239010 | 28.21 | 0.28 | **7.90** | SapurV1A.0935s0010.1 | Serine/Threonine-kinase |
| Stems | M1216190 | n/a | 0.00 | **8.30** | SapurV1A.1369s0020.1 | LRR receptor-like Serine/Threonine-kinase |
| Stems | M1399740 | 9.28 | 0.97 | **9.00** | SapurV1A.0021s0970.3 | serine/threonine-protein phosphatase |
| Stems | M1398290 | 36.67 | 0.27 | **9.90** | SapurV1A.0206s0300.1 | LRR receptor-like kinase |
| Stems | M1327161 | 1.59 | 6.30 | **10.00** | SapurV1A.0168s0220.1 | Serine/Threonine-kinase RIO1 |
| Stems | M1469140 | 34.29 | 0.35 | **12.00** | SapurV1A.1281s0010.1 | serine/threonine protein phosphatase 6 |
| Leaves | M1352440 | 1.30 | 10.00 | **13.00** | SapurV1A.0368s0330.1 | Serine/Threonine-kinase rio2 |
| Stems | M1431804 | 8.13 | 1.60 | **13.00** | SapurV1A.0629s0160.3 | LRR receptor-like kinase |
| Stems | M1262951 | 10.83 | 1.20 | **13.00** | SapurV1A.0206s0300.1 | LRR receptor-like kinase |
| Stems | M1339143 | 24.73 | 0.93 | **23.00** | SapurV1A.0960s0010.1 | LRR receptor-like kinase |
| Leaves | M1296640 | 1.16 | 25.00 | **29.00** | SapurV1A.0375s0170.8 | Serine/Threonine-kinase AFC3 |
| Buds | M1371350 | 1.27 | 26.00 | **33.00** | SapurV1A.0128s0280.2 | serine/threonine-protein phosphatase |
| Stems | M1456181 | 1.52 | 23.00 | **35.00** | SapurV1A.0113s0010.1 | LRR receptor-like kinase |
| Stems | M1445721 | 21.18 | 1.70 | **36.00** | SapurV1A.1199s0010.2 | LRR receptor-like kinase |
| Stems | M1392460 | 1.31 | 36.00 | **47.00** | tr\|M0RVD6 | Serine/threonine-protein phosphatase |
| Stems | M1426532 | 17.00 | 3.00 | **51.00** | SapurV1A.0236s0260.1 | LRR receptor-like kinase |
| Buds | M1436110 | 1.47 | 75.00 | **110.00** | SapurV1A.0039s0130.1 | LRR receptor-like kinase |
|  |  |  |  |  |  |  |
| *PPR* |  |  |  |  |  |  |
| Organ | Unique ID | fold change | Contaminated Mean fpkm | Non-contam Mean fpkm | *S. purpurea/prot ID* | Putative description |
| Stems | M1335820 | 7.47 | **62.00** | 8.30 | SapurV1A.0679s0140.1 | PPR containing plant protein |
| Stems | M1378023 | 1.59 | **35.00** | 22.00 | SapurV1A.0139s0140.1 | PPR containing plant protein |
| Stems | M1469492 | 1.21 | **23.00** | 19.00 | SapurV1A.0310s0120.1 | PPR containing plant protein |
| Stems | M1404460 | 1.10 | **22.00** | 20.00 | SapurV1A.0217s0190.1 | PPR containing plant protein |
| Stems | M1358240 | 1.35 | **12.00** | 8.90 | SapurV1A.0042s0260.1 | PPR containing plant protein |
| Stems | M1342392 | 1.00 | **11.00** | 11.00 | SapurV1A.1239s0040.1 | PPR containing plant protein |
| Stems | M1420190 | 2.32 | **8.60** | 3.70 | SapurV1A.0256s0250.2 | PPR containing plant protein |
| Stems | M1307350 | 1.03 | **6.20** | 6.00 | SapurV1A.0168s0150.1 | PPR containing plant protein |
| Stems | M1381040 | 1.02 | **4.20** | 4.10 | SapurV1A.0125s0400.1 | PPR containing plant protein |
| Stems | M1421614 | 1.46 | **1.90** | 1.30 | SapurV1A.0087s0250.1 | PPR containing plant protein |
| Stems | M1378660 | 1.83 | **1.70** | 0.93 | SapurV1A.1822s0020.1 | PPR containing plant protein |
| Stems | M1382312 | 1.67 | **1.40** | 0.84 | SapurV1A.0024s0280.1 | PPR containing plant protein |
| Stems | M1259130 | 1.92 | **0.69** | 0.36 | SapurV1A.0103s0260.1 | PPR containing plant protein |
| Buds | M1322491 | 3.44 | **0.62** | 0.18 | SapurV1A.0774s0050.1 | PPR containing plant protein |
| Stems | M1264500 | 1.75 | 0.57 | **1.00** | SapurV1A.0668s0200.1 | PPR containing plant protein |
| Leaves | M1376060 | 1.52 | 0.66 | **1.00** | SapurV1A.0040s0020.1 | PPR containing plant protein |
| Leaves | M1433531 | 1.59 | 0.69 | **1.10** | SapurV1A.0744s0100.1 | PPR containing plant protein |
| Leaves | M1285700 | 1.38 | 1.30 | **1.80** | SapurV1A.0128s0270.1 | PPR containing plant protein |
| Leaves | M1385220 | 1.50 | 1.20 | **1.80** | SapurV1A.0088s0170.1 | PPR containing plant protein |
| Stems | M1453780 | 4.08 | 0.49 | **2.00** | SapurV1A.1811s0020.1 | PPR containing plant protein |
| Stems | M1427452 | 3.14 | 0.70 | **2.20** | SapurV1A.0203s0270.1 | PPR containing plant protein |
| Leaves | M1422340 | 1.33 | 2.10 | **2.80** | SapurV1A.0368s0340.1 | PPR containing plant protein |
| Stems | M1251630 | 1.87 | 2.30 | **4.30** | SapurV1A.0735s0190.1 | PPR containing plant protein |
| Stems | M1414230 | 1.47 | 3.20 | **4.70** | SapurV1A.0399s0050.1 | PPR containing plant protein |
| Leaves | M1119581 | 1.28 | 3.90 | **5.00** | SapurV1A.0236s0140.1 | PPR containing plant protein |
| Stems | M1374900 | 1.79 | 2.80 | **5.00** | SapurV1A.0011s0680.1 | PPR containing plant protein |
| Leaves | M1438230 | 1.04 | 5.30 | **5.50** | SapurV1A.0203s0250.1 | PPR containing plant protein |
| Stems | M1412240 | 1.88 | 3.20 | **6.00** | SapurV1A.1384s0020.1 | PPR containing plant protein |
| Stems | M1469271 | 1.85 | 3.30 | **6.10** | SapurV1A.0735s0200.1 | PPR containing plant protein |
| Stems | M1435320 | 1.39 | 5.10 | **7.10** | SapurV1A.0161s0010.1 | PPR containing plant protein |
| Stems | M1296140 | 1.44 | 5.70 | **8.20** | SapurV1A.0076s0380.2 | PPR containing plant protein |
| Stems | M1299840 | 1.72 | 5.40 | **9.30** | SapurV1A.2292s0040.1 | PPR containing plant protein |
| Leaves | M1398654 | 1.27 | 11.00 | **14.00** | SapurV1A.0399s0090.1 | PPR containing plant protein |
| Stems | M1313510 | 1.73 | 8.10 | **14.00** | SapurV1A.0030s0400.1 | PPR containing plant protein |
| Stems | M1303700 | 42.42 | 0.33 | **14.00** | SapurV1A.0006s0080.1 | PPR containing plant protein |
| Stems | M1468760 | 1.73 | 11.00 | **19.00** | SapurV1A.1291s0010.1 | PPR containing plant protein |
| Leaves | M1414230 | 1.58 | 12.00 | **19.00** | SapurV1A.0399s0050.1 | PPR containing plant protein |
| Leaves | M1404460 | 1.11 | 18.00 | **20.00** | SapurV1A.0217s0190.1 | PPR containing plant protein |
| Stems | M1469910 | 1.11 | 19.00 | **21.00** | SapurV1A.0686s0050.2 | PPR containing plant protein |
| Stems | M1437091 | 2.94 | 8.50 | **25.00** | SapurV1A.0283s0210.1 | PPR containing plant protein |
| Stems | M1340250 | 1.76 | 17.00 | **30.00** | SapurV1A.0196s0210.1 | PPR containing plant protein |
| Stems | M1318810 | 1.29 | 24.00 | **31.00** | SapurV1A.0009s0700.1 | PPR containing plant protein |
| Leaves | M1305730 | 1.29 | 24.00 | **31.00** | SapurV1A.0161s0210.1 | PPR containing plant protein |
| Leaves | M1423130 | 1.05 | 41.00 | **43.00** | SapurV1A.0220s0420.1 | PPR containing plant protein |
| Stems | M1458852 | 2.46 | 35.00 | **86.00** | SapurV1A.0635s0030.1 | PPR containing plant protein |
